# Addressing the global challenge of bacterial drug resistance: insights, strategies, and future directions

**DOI:** 10.3389/fmicb.2025.1517772

**Published:** 2025-02-24

**Authors:** Arun Karnwal, Amar Yasser Jassim, Ameer Abbas Mohammed, Abdel Rahman Mohammad Said Al-Tawaha, Manickam Selvaraj, Tabarak Malik

**Affiliations:** ^1^Department of Microbiology, Graphic Era (Deemed to be University), Dehradun, India; ^2^Department of Marine Vertebrate, Marine Science Center, University of Basrah, Basrah, Iraq; ^3^Department of Biological Sciences, Al Hussein Bin Talal University Ma’an, Ma’an, Jordan; ^4^Department of Chemistry, Faculty of Science, King Khalid University, Abha, Saudi Arabia; ^5^Center of Bee Research and its Products (CBRP), and Unit of Bee Research and Honey Production, King Khalid University, Abha, Saudi Arabia; ^6^Department of Biomedical Sciences, Institute of Health, Jimma University, Jimma, Ethiopia

**Keywords:** antibiotic consumption, National Action Plans (NAPs), antibiotic resistance Bacteria (ARB), antibiotic-resistance genes (ARG), combination therapies, next-generation sequencing, resistance transmission, human health impacts

## Abstract

The COVID-19 pandemic underscored bacterial resistance as a critical global health issue, exacerbated by the increased use of antibiotics during the crisis. Notwithstanding the pandemic’s prevalence, initiatives to address bacterial medication resistance have been inadequate. Although an overall drop in worldwide antibiotic consumption, total usage remains substantial, requiring rigorous regulatory measures and preventive activities to mitigate the emergence of resistance. Although National Action Plans (NAPs) have been implemented worldwide, significant disparities persist, particularly in low- and middle-income countries (LMICs). Settings such as farms, hospitals, wastewater treatment facilities, and agricultural environments include a significant presence of Antibiotic Resistant Bacteria (ARB) and antibiotic-resistance genes (ARG), promoting the propagation of resistance. Dietary modifications and probiotic supplementation have shown potential in reshaping gut microbiota and reducing antibiotic resistance gene prevalence. Combining antibiotics with adjuvants or bacteriophages may enhance treatment efficacy and mitigate resistance development. Novel therapeutic approaches, such as tailored antibiotics, monoclonal antibodies, vaccines, and nanoparticles, offer alternate ways of addressing resistance. In spite of advancements in next-generation sequencing and analytics, gaps persist in comprehending the role of gut microbiota in regulating antibiotic resistance. Effectively tackling antibiotic resistance requires robust policy interventions and regulatory measures targeting root causes while minimizing public health risks. This review provides information for developing strategies and protocols to prevent bacterial colonization, enhance gut microbiome resilience, and mitigate the spread of antibiotic resistance.

## 1 Introduction

In recent years, antibiotics have made a significant contribution to socioeconomic growth by promoting healthcare, avoiding deaths, and increasing animal productivity. However, the inadequate use of antibiotics has exacerbated the emergence of bacterial resistance, failed treatments, increased disease rates, and increased healthcare expenses (Bunduki et al., 2024; Gulumbe et al., 2023; Watkins and Bonomo, 2020). In 2018, the global use of veterinary antibiotics was approximately 76,704 tons (Ardakani et al., 2024; de Lagarde et al., 2022; Zeedan et al., 2023), while medical antibiotic consumption was 14.3 defined daily doses (DDDs) per thousand people per day (Zhang R. M. et al., 2023). Antibiotic-resistant bacteria (ARB) account for over 25% of nosocomial infections, posing a growing challenge to healthcare systems. Projections suggest that by 2050, there will be a shocking 10 million deaths caused by these bacteria (Asmare et al., 2024). Reducing the use of antibiotics is crucial to prevent the spread of resistance in various contexts, such as healthcare facilities, animals, the food chain, and the environment (Bunduki et al., 2024). It will help to minimize the dangers to public health.

**Figure 1:**
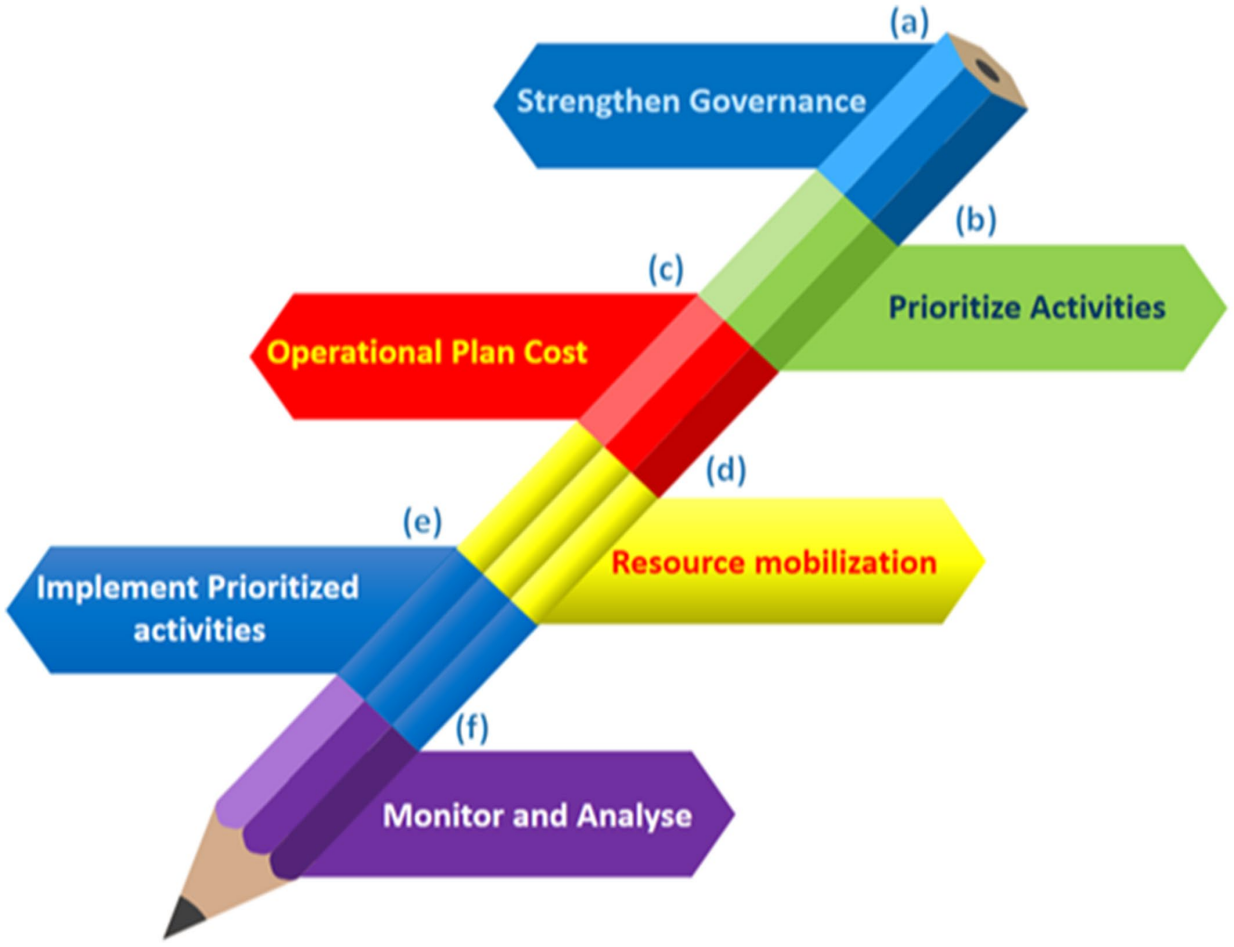
Six stages for the successful execution of NAPs on antimicrobial resistance in a sustainable manner. **(A)** Create an effective cross-sector coordination system and technical teams with defined roles, budget allocations, and accountability structures in place. **(B)** Conduct a consultative approach to select operations based on current conditions, resources, predicted impact, and feasibility. **(C)** Prioritize tasks, define responsibilities, dates, and locations, and incorporate existing financing streams into an operational plan. **(D)** Identify current and potential financiers, lobby to close the financing gap, and use indigenous funding through national initiatives and fiscal allocations when possible. **(E)** Work with internal and external stakeholders to sustainably implement prioritized initiatives. **(F)** Regularly evaluate and share progress and lessons from plan and endeavor execution.

The emergence of antimicrobial resistance in the environment, livestock, and humans increases the risk of human infection by resistant bacteria (Sachdeva et al., 2025). The human gut, rich in nutrients and maintaining an ideal temperature, fosters the spread of antibiotic resistance genes (ARGs) and antibiotic-resistant bacteria (ARB) due to its diverse microbiota (Das et al., 2022). ARGs and ARB within the human body pose a significant and growing health threat (Fang et al., 2023). This review analyzes global patterns in veterinary and clinical antibiotic use and presents National Action Plans (NAPs) (Figure 1) to combat antibiotic resistance. Additionally, it explores strategies for reversing resistance, reducing transmission post-colonization, and preventing the spread of resistant bacteria. Implementing these measures is crucial to mitigating antimicrobial resistance and its transmission to humans (Mendelson et al., 2024).

## 2 Challenges in global antibiotic usage and action plans

Although a recent global decline in antibiotic use for veterinary purposes, excessive antibiotic consumption in clinical settings remains a major concern, particularly in low- and middle-income countries (Alabi et al., 2024; O’Leary et al., 2024; Liu L. et al., 2021; Liu Y. et al., 2021). While many countries have implemented National Action Plans (NAPs) to combat antibiotic resistance, approaches vary significantly (Villarreal et al., 2023) (Table 1). The World Health Organization (WHO), the Centers for Disease Control and Prevention (CDC), and the European Centre for Disease Prevention and Control (ECDC) have raised alarms over the growing threat of antimicrobial resistance (AMR) (World Health Organization, 2024; Cobar and Cobar, 2024). WHO advocates for stricter regulations on antimicrobial use in animals, particularly those critical to human medicine, while the CDC and ECDC emphasize a One Health approach that integrates human, animal, and environmental health (Mercy et al., 2024; Catteau et al., 2024). These organizations call for reduced antibiotic use in animals, enhanced surveillance, and improved stewardship practices to curb the spread of resistant pathogens (Vekemans et al., 2021).

**Table 1:** Key challenges in global antibiotic usage and the limitations of current action plans in addressing antibiotic resistance (Cobar and Cobar, 2024; Berry, 2024; Bolbanabad et al., 2023; Khouja et al., 2022; Pham et al., 2024; Villarreal et al., 2023; Willemsen et al., 2022; Uddin et al., 2021).

Aspect	Description	Challenges	Contributing factors	Consequences	Proposed solutions
Global Antibiotic Usage	The overall utilization of antibiotics worldwide.	- Overuse and misuse of antibiotics leading to resistance. - Disparities in access to antibiotics between developed and developing countries. - Limited surveillance and regulation in some regions.	- Easy availability of antibiotics without prescription. - Lack of awareness among healthcare providers and patients regarding appropriate antibiotic use. - Economic factors influencing prescribing practices.	- Rising rates of antibiotic resistance. - Increased healthcare costs due to treatment failures and prolonged hospital stays. - Compromised effectiveness of existing antibiotics.	- Strengthening antibiotic stewardship programs globally. - Enhancing surveillance systems to monitor antibiotic use and resistance patterns. - Promoting education and awareness campaigns on appropriate antibiotic use.
Action Plans	Strategies and initiatives implemented by governments and organizations to address antibiotic resistance.	- Variation in the implementation and effectiveness of action plans across different regions. - Limited resources were allocated to execute the action plan. - Resistance to change among healthcare professionals and policymakers.	- Political and economic barriers to policy implementation. - Lack of coordination between healthcare sectors and stakeholders. - Insufficient investment in research and development of new antibiotics.	- Inadequate control over antibiotic resistance leading to public health crises. - Continued emergence and spread of multidrug-resistant pathogens. - Delayed innovation in antibiotic development due to market challenges.	- Strengthening regulatory frameworks to enforce antibiotic stewardship policies. - Increasing funding for research and development of new antibiotics and alternative therapies. - Fostering collaboration between governments, healthcare organizations, and pharmaceutical companies.
Global Surveillance	Monitoring and tracking antibiotic usage and resistance patterns worldwide.	- Inconsistent data collection and reporting methods. - Limited access to surveillance data, particularly in low-resource settings. - Challenges in standardizing surveillance metrics and definitions.	- Lack of investment in surveillance infrastructure and capacity building. - Reluctance among countries to share data due to concerns about confidentiality and sovereignty. - Fragmentation of surveillance efforts at the national and international levels.	- Incomplete understanding of global antibiotic resistance trends. - Difficulty in identifying emerging resistance threats and hotspots. - Suboptimal allocation of resources for targeted interventions.	- Establishing a standardized global surveillance network with harmonized data collection protocols. - Providing technical and financial support to enhance surveillance capabilities in low-resource settings. - Facilitating data sharing and collaboration through international partnerships and initiatives.
Antibiotic Development	Research and innovation in creating new antibiotics and alternative therapies.	- Decline in antibiotic discovery and development pipelines. - Regulatory hurdles and market challenges in bringing new antibiotics to market. - Limited investment in research for non-traditional antibiotic approaches.	- High failure rates and long timelines in antibiotic development. - Disincentives for pharmaceutical companies to invest in antibiotic R&D due to low profitability and uncertain returns. - Scientific and technical challenges in targeting drug-resistant pathogens.	- Fewer treatment options for drug-resistant infections. - Delayed availability of novel antibiotics for patients in need. - Potential resurgence of untreatable infections and pandemics.	- Implementing innovative funding models and incentives to stimulate antibiotic research and development. - Streamlining regulatory pathways and providing support for antibiotic clinical trials. - Investing in research on alternative approaches, such as phage therapy, immunotherapy, and antimicrobial peptides.
Public Awareness and Education	Informing and educating the public about antibiotic resistance and appropriate antibiotic use.	- Low awareness and understanding of antibiotic resistance among the general population. - Misconceptions and misinformation about antibiotics and their effectiveness. - Limited engagement of healthcare providers in patient education on antibiotic stewardship.	- Insufficient resources allocated for public health campaigns and educational initiatives. - Communication challenges in reaching diverse populations and marginalized communities. - Limited integration of antibiotic stewardship education into healthcare curricula.	- Continued overuse and misuse of antibiotics by patients and caregivers. - Delayed seeking medical care for infectious diseases due to misconceptions about antibiotics. - Resistance to behavior change and adherence to antibiotic treatment guidelines.	- Launching comprehensive public awareness campaigns on antibiotic resistance and prudent antibiotic use. - Engaging healthcare professionals as advocates for antibiotic stewardship and patient education. - Incorporating antibiotic stewardship education into school curricula and professional training programs.

### 2.1 Global antibiotic consumption trends in veterinary

In 2018, global veterinary antibiotic consumption was approximately 81,000 tons, with an estimated dosage of 75.16–82.56 mg per kilogram of animal weight (Sorbara et al., 2019; Sun et al., 2019). Ardakani et al. (2024) reported that in 2017, antimicrobial usage (AMU) in chickens, cattle, and pigs—comprising 93.75% of all food animals—amounted to 93,309 tonnes of active ingredients (Ardakani et al., 2024; Calayag et al., 2021). This figure is projected to rise by 11.5% to 104,079 tonnes by 2030, with pigs contributing the most to this increase (45%), followed by cattle (22%). In 2017, pigs consumed an average of 193 mg per population correction unit (PCU), while cattle had the lowest consumption at 42 mg/PCU (Rothrock et al., 2021).

**Figure 2:**
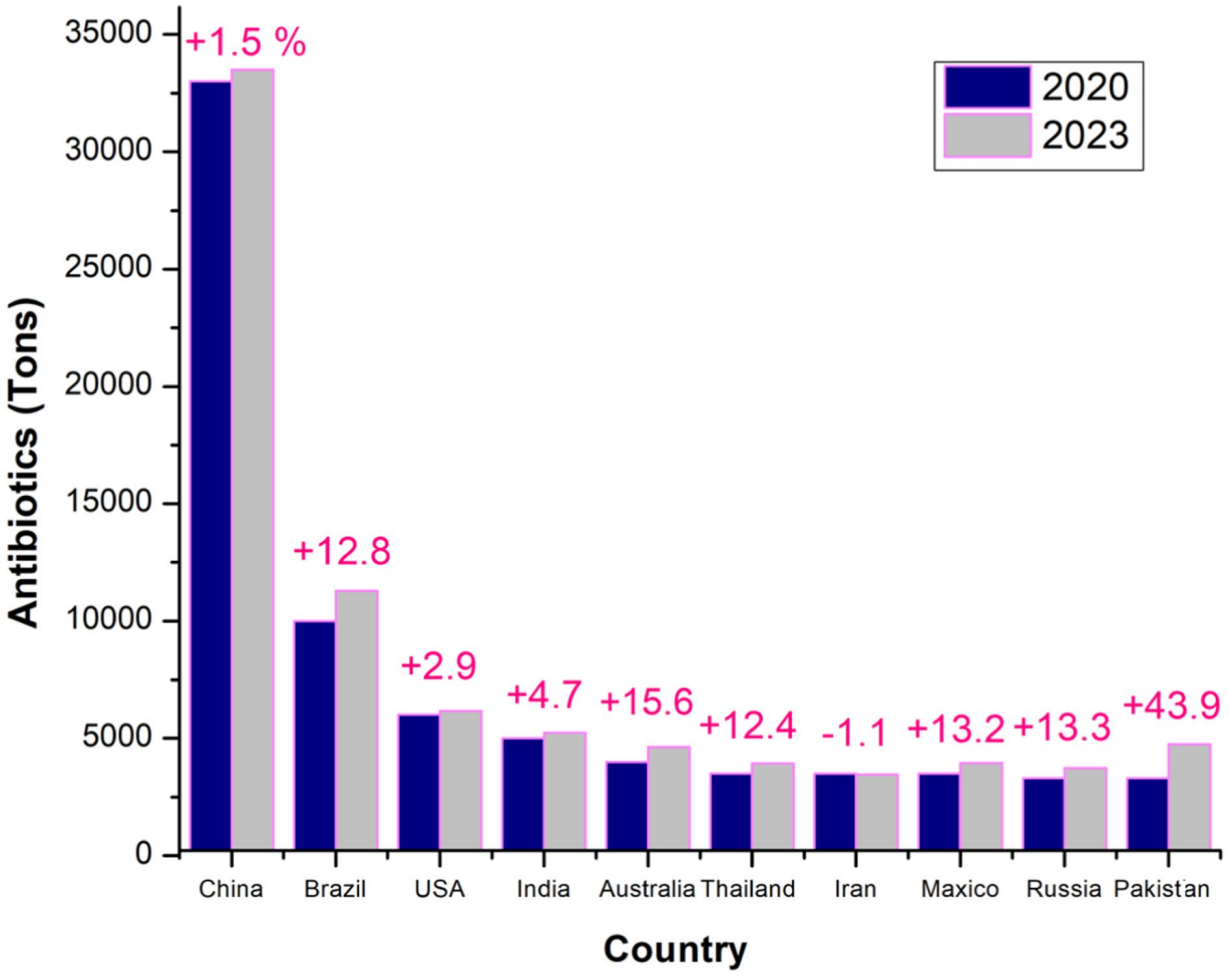
Global comparison of top antibiotic consumers in veterinary medicine: a detailed analysis of the leading countries in 2020 and 2023, highlighting trends in antibiotic usage and year variations (Ardakani et al., 2024).

Figure 2 illustrates trends in antibiotic usage across major countries over two-time points, highlighting shifts in consumption patterns and the rise in usage between the years under review. This comparison emphasizes the critical need for sustainable antibiotic use to combat antimicrobial resistance (AMR) (Ardakani et al., 2024; Al-Tawfiq et al., 2024). In 2017, chickens consumed an average of 68 mg/PCU, contributing to 33% of the global increase in AMU (Murray et al., 2021; Salam et al., 2023). Asia, the largest consumer of veterinary antibiotics in 2017, is expected to continue this trend, with its usage projected to grow by 10.3% by 2030, accounting for 68% of global usage by that time for all used antibiotics mentioned by Tiseo et al. (2020) and supported by Van Boeckel et al. (2015). Africa is forecast to experience the most significant increase, with an expected rise of 37% by 2030, although it will still account for just 6.1% of global consumption (Mercy et al., 2024). Meanwhile, Oceania, North America, and Europe are projected to see minimal growth in antimicrobial sales (Cheng et al., 2022; Iera et al., 2025). As the largest consumer in 2017, China is expected to maintain its position in 2030, with the top 10 consuming countries collectively accounting for 72% of global antimicrobial consumption by that year (Zuo et al., 2021) (see Tables 2,3).

**Table 2:** Trends in global antibiotic consumption from 2010 to 2023: an analysis of usage patterns across different regions and antibiotic (Al-Taani et al., 2022; Al Meslamani, 2023; Ardakani et al., 2024; Cuevas et al., 2021; Kamere et al., 2022; Quaik et al., 2020).

Year	Total antibiotic consumption (tons)	Consumption per animal (mg/kg)	Regional distribution of antibiotic usage (%)	Most used antibiotics (%)
2010	80,000	90.91–100.00	Asia (30%), Far East and Oceania (25%), Americas (20%)	Tetracyclines, Penicillins
2011	82,000	88.65–96.35	Asia (32%), Far East and Oceania (24%), Americas (18%)	Tetracyclines, Penicillins
2012	85,000	85.12–92.73	Asia (34%), Far East and Oceania (23%), Americas (17%)	Tetracyclines, Penicillins
2013	88,000	82.89–90.55	Asia (35%), Far East and Oceania (22%), Americas (16%)	Tetracyclines, Penicillins
2014	90,000	80.88–88.63	Asia (36%), Far East and Oceania (21%), Americas (15%)	Tetracyclines, Penicillins
2015	92,000	79.13–86.96	Asia (37%), Far East and Oceania (20%), Americas (14%)	Tetracyclines, Penicillins
2016	85,000	78.33–86.33	Asia (36%), Far East and Oceania (20%), Americas (15%)	Tetracyclines, Penicillins
2017	83,000	76.74–84.44	Asia (35%), Far East and Oceania (21%), Americas (16%)	Tetracyclines, Penicillins
2018	81,000	75.16–82.56	Asia (34%), Far East and Oceania (22%), Americas (17%)	Tetracyclines, Penicillins
2019	79,000	73.81–81.33	Asia (33%), Far East and Oceania (23%), Americas (18%)	Tetracyclines, Penicillins
2020	78,000	72.55–80.33	Asia (32%), Far East and Oceania (24%), Americas (19%)	Tetracyclines (34%), Penicillins (13%)
2021	77,000	71.37–79.22	Asia (31%), Far East and Oceania (25%), Americas (20%)	Tetracyclines (35%), Penicillins (14%)
2022	76,000	70.25–78.17	Asia (30%), Far East and Oceania (26%), Americas (21%)	Tetracyclines (35%), Penicillins (14%)
2023	75,000	69.19–77.17	Asia (29%), Far East and Oceania (27%), Americas (22%)	Tetracyclines (36%), Penicillins (15%)

**Table 3:** Global antibiotic consumption trends by class from 2010 to 2023: a comparative analysis of usage patterns across different antibiotic classes (Al-Taani et al., 2022; Al Meslamani, 2023; Ardakani et al., 2024; Cuevas et al., 2021; Kamere et al., 2022; Quaik et al., 2020).

Year	Total antibiotic consumption (tons)	T*	P*	M*	S*	A*	F*	O*
2010	80,000	40%	20%	15%	10%	5%	5%	5%
2011	82,000	38%	22%	16%	11%	6%	4%	3%
2012	85,000	37%	23%	17%	12%	6%	4%	3%
2013	88,000	36%	24%	17%	12%	6%	4%	3%
2014	90,000	35%	25%	18%	12%	6%	4%	3%
2015	92,000	34%	26%	18%	12%	6%	4%	3%
2016	85,000	33%	27%	19%	12%	6%	4%	3%
2017	83,000	32%	28%	19%	12%	6%	4%	3%
2018	81,000	32%	29%	19%	12%	6%	4%	3%
2019	79,000	31%	30%	20%	11%	6%	4%	3%
2020	78,000	30%	31%	20%	11%	6%	4%	3%
2021	77,000	30%	32%	20%	11%	6%	4%	3%
2022	76,000	29%	33%	21%	10%	6%	4%	3%
2023	75,000	28%	34%	21%	10%	6%	4%	3%

*T: tetracyclines, P: penicillins, M: macrolides, S: sulfonamides, A: aminoglycosides, F: fluoroquinolones, and O: others.

Tetracyclines and penicillins were the primary antibiotics used in animal health, accounting for 40.5 and 14.1% of total consumption, respectively (Ardakani et al., 2024). However, discrepancies in antimicrobial usage (AMU) data, particularly from China, highlight the need for further investigation into the accuracy of reported figures. Between 2015 and 2030, human AMU is projected to increase by 15%, paralleling the rising demand for food animals (Cherian et al., 2023). Interestingly, the expected surge in animal antimicrobial consumption is lower than previous estimates—Van Boeckel et al. (2019) had predicted a 53% rise by 2030. These discrepancies stem from variations in data sources, specifically in China, where recent reports from the Ministry of Agriculture indicate a significant decline in AMU, raising concerns about data reliability (Zhang K. et al., 2022). However, The fourth JIACRA report analyzed antimicrobial consumption (AMC) and antimicrobial resistance (AMR) trends across 2014–2021, based on data from EU surveillance networks. In 2021, the total AMC for humans was 125.0 mg/kg, while for food-producing animals, it was 92.6 mg/kg. Over this period, AMC in animals decreased by 44%, while human consumption remained stable (European Centre for Disease Prevention and Control (ECDC), 2024). Positive associations between AMC and AMR in both sectors were observed, indicating the influence of AMC on resistance patterns. The report also highlighted that reductions in AMC in both humans and animals were often associated with improved antimicrobial susceptibility in bacteria. These trends suggest that efforts to reduce AMC have been effective in many countries, although further actions are needed to maintain and strengthen these gains (European Centre for Disease Prevention and Control (ECDC), 2024). Measures such as vaccination and improved hygiene are crucial in reducing reliance on antimicrobials and promoting health.

In high-income countries (HICs), antimicrobial sales have declined due to stewardship programs promoting responsible antibiotic use (Ahmed et al., 2024). The United Kingdom and the United States, for instance, have successfully reduced veterinary antimicrobial use through targeted resistance strategies and stricter guidelines (Hawkins et al., 2022; Sachan et al., 2023; Ahmed et al., 2024). However, trends have been inconsistent across regions. Canada initially saw a reduction in antimicrobial sales but later experienced an increase. Globally, clinical antibiotic usage has risen, with daily defined doses (DDDs) per 1,000 people increasing from 9.8 in 2000 to 14.3 in 2018 (Browne et al., 2021). This trend raises serious concerns about the growing prevalence of antibiotic-resistant bacteria, which pose a significant public health threat.

A notable shift in global antibiotic consumption occurred during the COVID-19 pandemic. Between 2019 and 2020, antibiotic utilization dropped by 20.84%, from 2,928.33 units per 1,000 individuals to 2,317.94 units (Khouja et al., 2022). This decline coincided with pandemic-related control measures, suggesting a correlation between stricter healthcare protocols and reduced antibiotic use. Additionally, hospitalized patients exhibited a shift from using antibiotics in the “Access” category to more restrictive “Watch” and “Reserve” groups, reflecting changes in prescribing patterns (Hussein and Ali, 2021; Hussein et al., 2022).

Paradoxically, the early stages of the pandemic saw a surge in antimicrobial consumption, driven by concerns over bacterial co-infections and the precautionary use of antibiotics, despite COVID-19 being a viral disease (Kumar et al., 2023). Over time, stricter regulations led to a global decline in antibiotic consumption. However, the overuse and misuse of antibiotics during the pandemic exacerbated antimicrobial resistance (AMR), heightening the risk of treatment failure, prolonged hospital stays, and increased mortality (Hussein et al., 2022). The pandemic underscored the urgent need for stronger antibiotic stewardship, responsible prescribing practices, and enhanced global awareness to mitigate the threat of AMR. Without immediate intervention, AMR could undermine modern medicine, making once-treatable infections life-threatening (Gulumbe et al., 2023).

### 2.2 Challenges in implementing global action plans for antimicrobial resistance

The Global Action Plan on Antimicrobial Resistance (AMR), endorsed by the 68th World Health Assembly in 2015, aimed to drive global efforts to combat antibiotic resistance (Gostin et al., 2020; Truché et al., 2020). Since then, numerous governments and regions have developed strategic initiatives to address AMR (Khouja et al., 2022; Bolbanabad et al., 2023). The AMR National Action Plan (NAP) Library was assessed on January 31, 2023 (Saleem et al., 2022), revealing that 134 out of 194 countries—comprising 69% of nations worldwide, including 40 non-English-speaking countries—have officially adopted NAPs. While most nations align with the Global Action Plan (GAP) framework, significant variations exist in the implementation of AMR programs (Charani et al., 2023; Chua et al., 2021). According to the World Organization for Animal Health (WOAH), approximately one-third of countries globally continued using antibiotics for livestock growth promotion in 2022 (Gehring et al., 2023). These disparities reflect inconsistencies in the design, execution, and monitoring of NAPs across different regions. The necessity of a well-structured, country-specific NAP tailored with targeted interventions to combat AMR is indisputable (Charani et al., 2023).

However, concerns persist regarding the effectiveness of current policies, particularly in low- and middle-income countries (LMICs), where AMR remains a pressing issue (Bolbanabad et al., 2023). Willemsen et al. (2022) emphasize that inadequate infrastructure, a lack of skilled professionals, and limited financial resources hinder the successful implementation of AMR management strategies. Furthermore, the increased interconnection between human populations, livestock, and agricultural ecosystems in LMICs exacerbates the risk of antibiotic resistance transmission, highlighting the urgent need to bridge these gaps (Checcucci et al., 2020) (see Table 4).

**Table 4:** A comprehensive overview of the key challenges, disparities, and barriers in the effective implementation of global action plans on antimicrobial resistance (AMR): A comparative analysis across regions and stakeholders (Anderson et al., 2019; Ashiru-Oredope et al., 2023; Bolbanabad et al., 2023; Willemsen et al., 2022; Chinemerem Nwobodo et al., 2022; Pham et al., 2024; Villarreal et al., 2023).

Challenges and disparities	Description	Implications
Lack of coordination	Limited coordination among countries and stakeholders in implementing GAPs leads to fragmented efforts and inefficiencies.	Inconsistent strategies and priorities hinder the effectiveness of AMR containment measures.
Resource constraints	Many countries, particularly low- and middle-income nations, lack adequate financial resources, infrastructure, and trained personnel to implement GAPs effectively.	Inadequate funding and infrastructure result in suboptimal surveillance, stewardship, and infection prevention efforts.
Limited surveillance capacity	Weak surveillance systems in some regions result in underreporting and insufficient data on AMR prevalence and trends.	Inaccurate data impedes evidence-based decision-making and monitoring of AMR containment efforts.
Inadequate access to essential medicines	Disparities in access to antimicrobials and diagnostics contribute to inappropriate use and misuse of antibiotics, fueling AMR.	Limited access to effective treatments jeopardizes patient outcomes and undermines efforts to control resistant infections.
Poor antibiotic stewardship practices	Inadequate implementation of antimicrobial stewardship programs in healthcare facilities leads to overuse and misuse of antibiotics.	Suboptimal prescribing practices contribute to the emergence and spread of resistant pathogens, compromising patient safety.
Antibiotic use in agriculture	The widespread use of antibiotics in agriculture, particularly for growth promotion and prophylaxis, contributes to AMR through environmental contamination and selection pressure.	Agricultural practices contribute significantly to AMR but are often inadequately addressed in GAPs.
Limited regulatory oversight	Weak regulatory frameworks and enforcement mechanisms fail to effectively regulate the sale and use of antibiotics in human and veterinary medicine.	Inconsistent regulations enable the unrestricted use of antibiotics, exacerbating AMR.
Global disparities in AMR awareness	Variations in AMR awareness among policymakers, healthcare providers, and the general public hinder coordinated action and behavior change.	Inadequate awareness perpetuates misconceptions about antibiotic use and resistance, hindering efforts to address AMR.
Inequitable access to technology and innovation	Disparities in access to diagnostic tools, vaccines, and novel antimicrobial agents limit effective AMR management in resource-limited settings.	Lack of access to innovative solutions impedes progress in controlling resistant infections and improving patient outcomes.

In 2019, agricultural employment accounted for an average of 23.51% of the global workforce, but this figure concealed stark contrasts between high- and low-income nations. In wealthier countries, where economies have shifted toward industry and services, agricultural employment was typically below 5%. In contrast, it exceeded 70% in many low- and middle-income nations, underscoring agriculture’s role as a primary livelihood source (Chen et al., 2023). The unregulated livestock trade exacerbates global challenges, particularly antibiotic resistance. In Colombia (2018), the widespread presence of cattle, along with the illegal sale of refrigerated chicken meat in Nigeria, illustrates how weak regulation enables the spread of resistant bacteria (Zapata-Cortés, 2020). Many countries attempt to mitigate environmental resistance through top-down strategies, such as Non-Aligned Party interventions. However, these measures often fall short in moderate- and low-income nations due to systemic barriers, including a shortage of skilled professionals, weak veterinary drug regulations, illegal livestock trade, and frequent human-animal interactions (Iera et al., 2025; Anderson et al., 2019). Effectively combating antibiotic resistance requires comprehensive national action plans that integrate both top-down policies and grassroots initiatives. While strong regulations and interventions are crucial for promoting responsible antibiotic use, community-driven strategies—such as public awareness campaigns and local management efforts—are equally vital, particularly in developing regions where agriculture remains central to the economy and infrastructure is limited (see Table 5).

**Table 5:** A comprehensive overview of global efforts to combat antimicrobial resistance, highlighting common challenges and disparities across regions and countries (Catteau et al., 2024; Charani et al., 2023; Chua et al., 2021; Özçelik et al., 2022; Tanno and Demoly, 2020).

Action plan name	Year implemented	Main focus areas	Challenges in implementation	Disparities in implementation
WHO GLOBAL Action Plan	2015	Surveillance, Awareness, Research	Limited surveillance infrastructure, lack of awareness among healthcare providers	Disparities in funding and resources between developed and developing countries
US National Action Plan	2015	Antimicrobial Stewardship	Resistance from agricultural and pharmaceutical industries, lack of regulatory power	Variations in implementation and enforcement across states
UK 5-Year Antimicrobial Strategy	2013	Education, Innovation	Resistance from prescribing physicians, funding limitations for research and education	Regional variations in awareness and access to antimicrobial resources
EU One Health Action Plan	2017	One Health Approach	Coordination challenges between human and veterinary healthcare sectors	Disparities in regulatory frameworks and surveillance capabilities
Australian National AMR Strategy	2015	Surveillance, Education	Limited data sharing between states and territories, inadequate public awareness	Rural and remote regions face challenges in accessing healthcare.

## 3 Sources and transmission pathways of antibiotic resistance in medical and livestock environments

Previous studies (Checcucci et al., 2020; Hetman et al., 2022; Osman et al., 2021) have identified healthcare facilities, farmland, wastewater treatment plants (WWTPs), and agricultural areas as key sources of ARGs and ARB. These regions serve as significant reservoirs of resistant bacteria and genes, facilitating the transmission of bacterial resistance among animals, humans, and the environment (Bunduki et al., 2024; Mugerwa et al., 2021; Okpala et al., 2021). Consequently, they are crucial in antibiotic stewardship and resistance prevention efforts. Hospitals, in particular, are significant contributors to the spread of ARB and ARG (Haseeb et al., 2022). Table 6 provides a comprehensive overview of the sources and transmission pathways of antibiotic resistance in medical and livestock settings. It details various sources, including freshwater bodies, WWTPs, farms, hospitals, and their respective transmission mechanisms. Understanding these pathways is essential for developing effective strategies to curb the spread of antibiotic resistance.

**Table 6:** Sources and transmission pathways of antibiotic resistance in various environments (Acolatse et al., 2022; Harun et al., 2024; Haseeb et al., 2022; Liu et al., 2018; Otieku et al., 2024).

Environment	Sources of antibiotic resistance	Transmission Pathways
Hospitals	- Contaminated surfaces and objects	- Direct contact with contaminated surfaces and objects
	- Aerosols carrying pathogenic bacteria and ARG	- Inhalation of aerosols generated in hospital environments
	- Use of antibiotics leading to ARB in hospital settings	- Interaction with contaminated objects or patients
Farms	- Widespread use of veterinary antibiotics	- Soil and water contamination with ARB and ARG
	- High concentrations of animals	- Direct contact between animals and humans
	- Nutrient-rich environments fostering bacterial growth	- Consumption of contaminated food products
Wastewater Treatment Plants (WWTPs)	- Presence of ARG in wastewater	- Discharge of treated wastewater into the environment
	- Inefficiencies in conventional treatment processes	- Aerosols generated during treatment processes
	- Transmission from municipal and hospital effluents	- Irrigation of agricultural fields with treated wastewater
	- Exposure to ARG and ARB through contaminated produce and water	- Direct contact with contaminated irrigation water
Freshwater Bodies	- Pollution from urban, industrial, and agricultural runoff	- Recreational activities in contaminated water bodies
	- Presence of ARG and ARB in aquatic environments	- Inhalation of aerosols from contaminated water bodies
	- Transmission via direct contact with pets and shared transportation systems	- Ingestion of contaminated water and food products

Hospitals, due to their specialized functions, inevitably serve as reservoirs for infectious agents. Various healthcare environments, including water systems (sewers, taps, sinks), medical instruments (scissors, work tables, switches, infusion stands), and bedding (mattresses), are highly susceptible to microbial contamination and biofilm formation. The presence of antibiotic-resistant genes (ARGs) and antibiotic-resistant bacteria (ARBs) significantly increases the risk of pulmonary infections (Haseeb et al., 2022). Research by He et al. (2020) confirms that hospital bacteria commonly exhibit antibiotic resistance, reinforcing the role of healthcare facilities as hubs for ARG and ARB transmission. Additionally, exposure to pharmaceuticals, contaminated surfaces, and shared hospital wards facilitates ARB spread among healthcare workers and patients. Mitigating contamination requires strict antibiotic stewardship, rigorous sterilization protocols, and standardized medical waste management.

Similarly, agricultural environments—particularly farms with intensive veterinary antibiotic use, high animal densities, and nutrient-rich conditions—are hotspots for antibiotic resistance emergence and proliferation (Bai et al., 2022). Studies (Bai et al., 2022; Cui et al., 2024; Ding et al., 2022; Georgakakos et al., 2021) confirm the widespread presence of ARGs and ARBs in animal waste, wastewater, soil, and air. Resistance genes such as tet(X) (conferring tigecycline resistance) and mcr-1 (associated with colistin resistance) have been detected in humans, animals, and the environment (Khan et al., 2021). Additionally, resistant bacterial strains—including extended-spectrum beta-lactamase (ESBL)-producing *E. coli* and methicillin-resistant *Staphylococcus aureus* (MRSA)—have been identified in agricultural settings (Crespo-Piazuelo and Lawlor, 2021). Environmental factors such as antibiotics, pH, temperature, and oxygen levels facilitate ARG transfer between bacteria, contributing to cross-contamination risks for agricultural workers and surrounding communities (Carfora et al., 2016; Liu et al., 2016; Tong et al., 2022; Hounmanou et al., 2021).

Wastewater treatment plants (WWTPs), processing municipal and hospital effluents, are also major reservoirs of ARBs and ARGs (Bueno et al., 2020). Studies have detected ARGs such as *sulI, qnrA, ermB*, and *blaCMY* in wastewater (Herraiz-Carboné et al., 2022; Kayali and Icgen, 2020), highlighting the limitations of conventional treatment methods. Aerosols generated during wastewater processing can disperse ARGs into surrounding areas, while irrigation with contaminated water and agricultural use of animal waste further propagate their spread into food products (Liu and Song, 2019). Consequently, fresh produce and meat remain vulnerable to contamination throughout production and processing, posing potential health risks to consumers.

Freshwater bodies are similarly at risk of ARG and ARB contamination from urban and industrial discharges, as well as agricultural runoff. Human exposure occurs through direct contact during recreational activities, inhalation of contaminated aerosols, or ingestion of polluted water (Che et al., 2019). Additionally, close interactions between pet owners and animals, along with shared public spaces, may contribute to ARG and ARB dissemination. However, accurately assessing human exposure remains challenging due to the complexity and variability of these transmission pathways.

## 4 Interventions for mitigating antibiotic resistance transmission in humans

ARG and ARB in the human body pose substantial health hazards comparable to ticking time bombs. When someone is sick or has a weaker immune system, opportunistic infections can quickly multiply, which can cause antibiotics not to work effectively (Andrina et al., 2020). ARGs and ARB can arise within the human body through two primary mechanisms. The first mechanism involves *de novo* emergence via spontaneous mutation in the host microbiome or by introducing external ARBs and ARGs (Alame Emane et al., 2021). This can be colloquially described as “starting from scratch.” The second mechanism involves horizontal gene transfer (HGT) of ARGs within bacterial communities, a process denoted as “from less to more” (Zhang F. et al., 2022).

**Figure 3:**
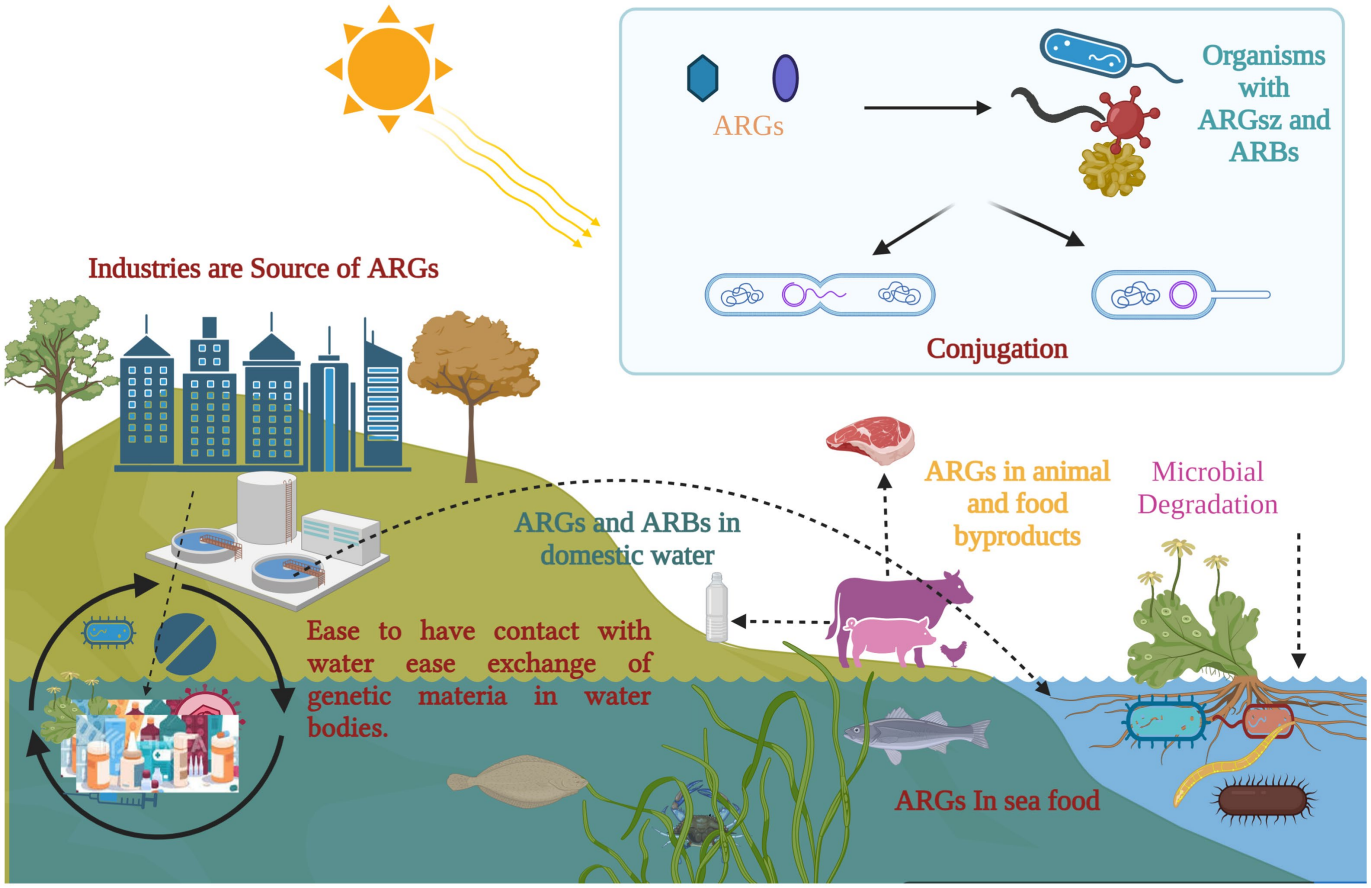
Antimicrobial resistance and its potential implications across organisms: exposure to selective pressure can promote the transmission of antibiotic resistance genes (ARGs) within microbial communities, including biofilms. Floating vegetation has the ability to absorb and harbor ARGs, which are introduced into aquatic environments from various sources. Once in these environments, ARGs can spread through microbial genetic exchange, food chains, and selective pressure. A potential pathway for ARGs includes transmission to humans and animals through contaminated water or infected fish. (Created with BioRender.com).

Moreover, it is essential to have practical approaches for restoring the sensitivity of ARB to prevent the emergence of additional resistance. Therefore, it is crucial to hinder the colonization of foreign ARG and ARB in humans, reduce the horizontal gene transfer (HGT) of the collection of resistance genes within the body, and reverse bacterial resistance (see Figure 3).

### 4.1 ARB transmission and bacterial colonization dynamics

According to Salyers et al. (2004) and Lee et al. (2020), antibiotic-resistant bacteria (ARB) play a crucial role in transferring antibiotic resistance genes (ARGs) to other organisms, including commensal bacteria in the intestines. Pathogenic bacteria have developed various virulence factors to overcome colonization resistance, allowing them to establish themselves in the gut and cause infection or disease. These factors significantly impact the human microbiome.

**Figure 4:**
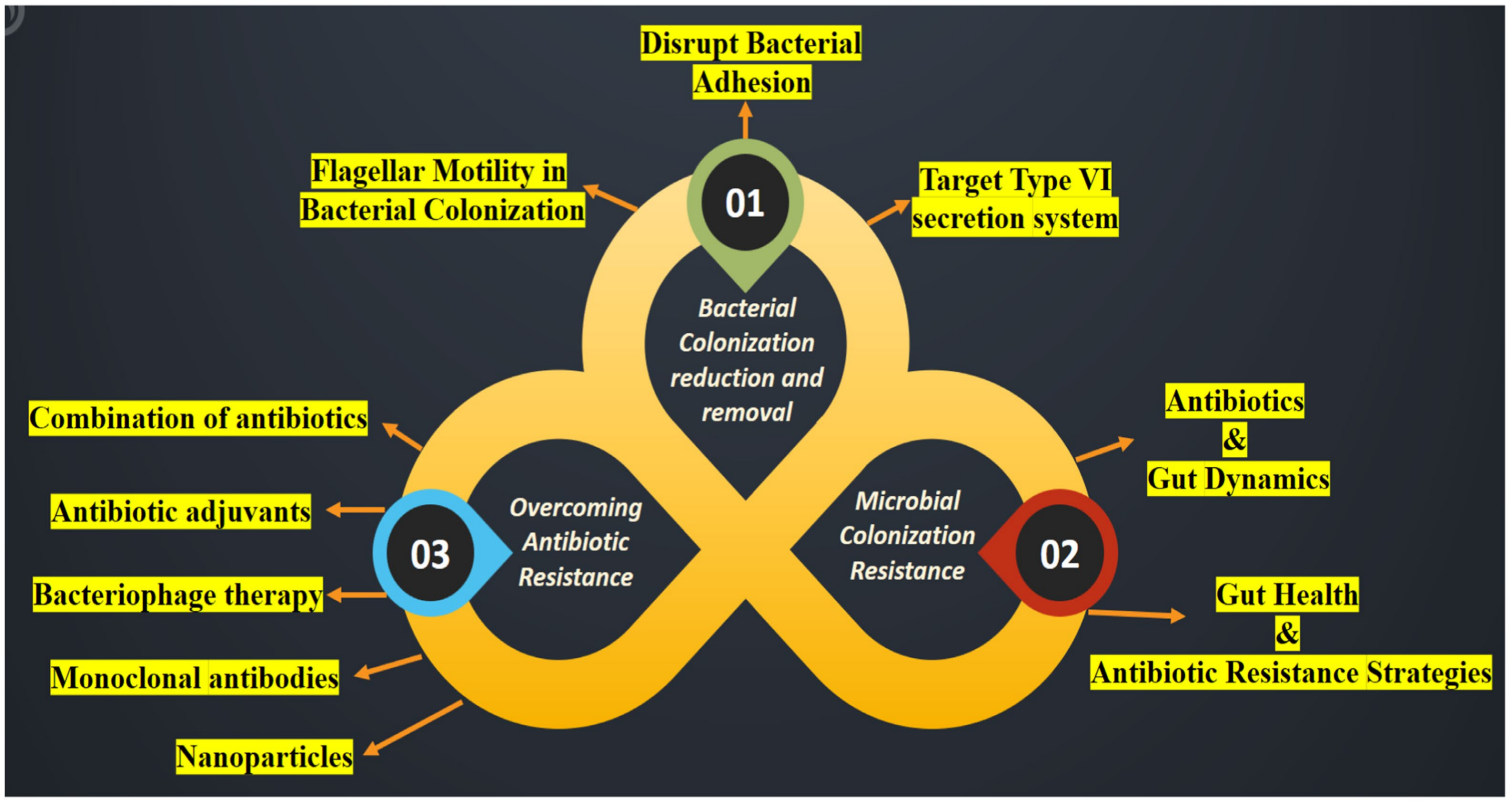
An illustration highlighting the suppression of colonization and multiplication by ARB and Antibiotic-Resistant Genes (ARG). Strategies to hinder the spread of the pathogen encompass the following: (01) reducing the ability of bacteria to colonize the microbiome; (02) strengthening resistance to colonization by the microbiome and preventing horizontal gene transfer; and (03) reversing bacterial resistance to antibiotics.

Bacterial colonization is a complex process involving movement, response to chemical signals, attachment, penetration, and the use of a specialized secretion system known as the Type VI secretion system (T6SS) (Klassert et al., 2021) (Figure 4). Table 7 presents a detailed overview of strategies to reduce bacterial colonization capacity by targeting flagellar motility, disrupting bacterial adhesion, and leveraging the Type VI secretion system. Each strategy is supported by examples of methods or approaches for effective implementation (Borgeaud et al., 2015).

**Table 7:** Strategies to combat bacterial colonization: targeting motility, adhesion, and secretion mechanisms (Ducarmon et al., 2019; Hsieh et al., 2019; Klassert et al., 2021; Klassert et al., 2020).

Strategy	Description	Examples/Methods
Targeting Flagellar Motility	Flagellar motility is crucial for bacterial movement and colonization. Inhibiting flagellar function can impede bacterial migration and adhesion to host surfaces.	1. Chemical inhibitors: Compounds that interfere with flagellar motor proteins or disrupt flagellar assembly.
		2. Genetic manipulation: Knocking out genes involved in flagellar synthesis or assembly can impair flagellar motility.
		3. Physical barriers: Surface coatings or materials that inhibit flagellar movement by creating an unfavorable environment for bacterial attachment.
		4. Antibodies: Targeting flagellar proteins with specific antibodies can prevent bacterial motility and adhesion.
Disrupting Bacterial Adhesion	Bacterial adhesion is a critical step in colonization. Disrupting adhesion mechanisms prevents bacteria from attaching to host tissues and surfaces, reducing colonization capacity.	1. Adhesion inhibitors: Compounds that interfere with bacterial adhesins or host receptors, preventing attachment.
		2. Anti-adhesion vaccines: Vaccines containing antigens from bacterial adhesins can induce an immune response, blocking bacterial attachment.
		3. Antibodies: Monoclonal antibodies targeting bacterial adhesins can block adhesion to host cells.
		4. Competitive inhibitors: Molecules that compete with bacteria for binding sites on host surfaces, preventing colonization.
Type VI Secretion System (T6SS)	The T6SS is a bacterial weapon used for interbacterial competition and host colonization. Inhibiting T6SS activity can disrupt bacterial colonization and virulence.	1. Small molecule inhibitors: Compounds that target T6SS components or disrupt T6SS function, impairing bacterial competition and colonization.
		2. Genetic manipulation: Knocking out or downregulating T6SS genes in bacteria can attenuate virulence and colonization capacity.
		3. Phage therapy: Bacteriophages targeting T6SS-positive bacteria can selectively kill pathogens, reducing colonization in the host.
		4. Immunotherapy: Neutralizing antibodies against T6SS effectors or structural proteins can block T6SS-mediated toxicity and colonization.

#### 4.1.1 Targeting flagellar motility for bacterial colonization prevention

**Figure 5:**
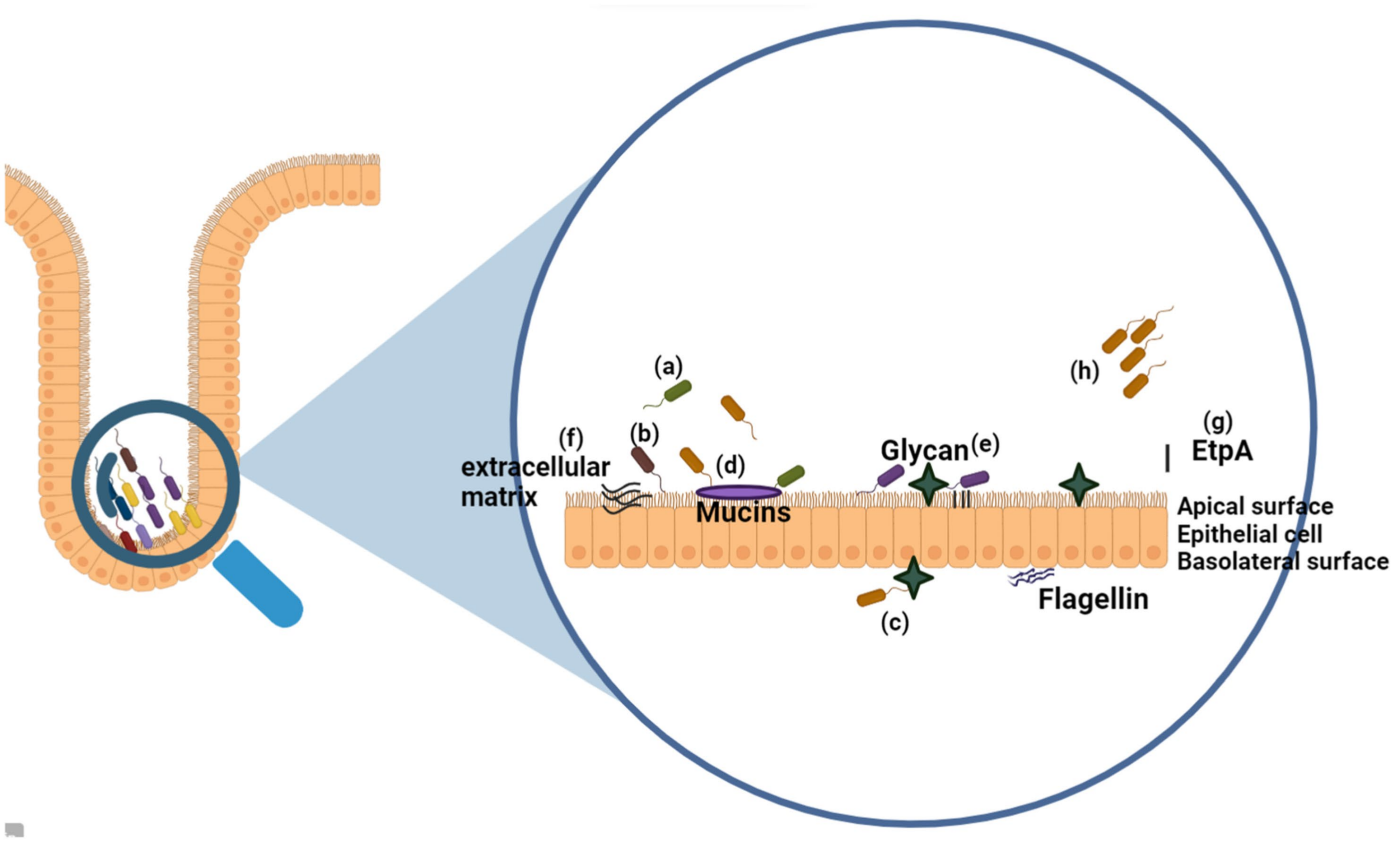
Overview of the Bacterial Flagellum as an Adhesin (a) The flagellum facilitates bacterial attachment to eukaryotic cells indirectly by enabling motility, allowing bacteria to reach target sites. (b) It can also directly bind to epithelial cells on both the apical and basolateral surfaces. (c) The flagellum targets various receptors, including mucus, mucins, glycans on cell surfaces or in mucus, extracellular matrix (ECM) proteins, and the bacterial-secreted protein EtpA, which aids in adhesion to host cells. (d) Furthermore, the flagellum can mediate inter-bacterial adhesion, connecting different bacterial species. (Created with BioRender.com).

Flagellar motility and chemotaxis play crucial roles in helping bacteria detect nutrient sources and thrive in favorable environmental conditions, enhancing their colonizing ability (Colin et al., 2021). The bacterial flagellum is a remarkable molecular machinery that facilitates bacterial or pathogenic movement. Through flagellar-mediated chemotaxis (Colin et al., 2021), bacteria can navigate toward specific regions within new hosts, thereby promoting colonization, invasion, and growth (Figure 5). Recent research by Kreuder et al. (2020) demonstrated that a short noncoding RNA, CjNC110, can influence the mobility of *Campylobacter jejuni*, enhancing its ability to establish in animal hosts. Consequently, modulating flagellar mobility may reduce bacterial adherence and motility.

Additionally, studies by Šimunović et al. (2020) have shown that essential oils and ethanolic extracts can diminish *Campylobacter jejuni* invasion and motility on INT407 epithelial cells by targeting the LuxS system. Given the significance of flagellar motility and chemotaxis in bacterial colonization, interventions aimed at reducing bacterial or pathogen motility hold promise for colonization prevention. Bacteria with decreased motility are less likely to locate favorable environmental niches, limiting their ability to adhere to and penetrate host tissues (Colin et al., 2021).

#### 4.1.2 Mechanisms and strategies to disrupt bacterial adhesion

To establish colonization and cause infection, *Staphylococcus aureus* must first adhere to and invade host tissues (Asadi et al., 2019). This process is facilitated by specific surface proteins known as adhesins, which play a crucial role in bacterial attachment. Among these, fibronectin-binding proteins (FnBPA and FnBPB) and laminin-binding proteins (Lmb) are particularly significant, aiding in persistent colonization of the nasal passages and intestines (Tewawong et al., 2020; Speziale and Pietrocola, 2020). Additionally, clumping factors A and B (ClfA and ClfB) enhance adhesion by binding to skin proteins, further promoting nasal colonization (Chen et al., 2020).

For successful infection, pathogens must firmly attach to the mucosal epithelium while overcoming host defenses such as secretions, movement, and peristalsis. Bacterial pili, encoded by multiple genes, are critical in this process, enhancing survival and biofilm formation. Type IV pili (T4P) are particularly essential for bacterial attachment, niche selection, and population expansion (Ligthart et al., 2020). Preventing bacterial adhesion can significantly reduce colonization and infection risk, strengthening the host’s ability to combat pathogens. Various strategies have been explored to disrupt bacterial attachment, including inhibiting pili formation, interfering with adhesion mechanisms, and utilizing vaccines or antibodies targeting adhesion factors (O'Brien et al., 2002).

One promising approach involves lactoferrin, which exhibits antibacterial properties by downregulating virulence genes associated with adhesion, such as flagellin, F18 fimbriae, and F4 fimbriae in intestinal epithelial cells (Dierick et al., 2020). Similarly, Ortiz et al. (2021) demonstrated that rifaximin, oregano extract, and carvacrol can modulate the expression of adhesion-related genes (ae, aggA, aap, pic, and aggR) in *Escherichia coli*, reducing its ability to adhere to HEp-2 cells. These findings offer promising insights into the molecular mechanisms governing bacterial colonization and highlight potential pathways for developing targeted anti-adhesion vaccines.

Further research by Von Mentzer et al. (2020) identified glycosphingolipids as potential receptors for enterotoxigenic *E. coli* (ETEC) colonization factor CS30. Additionally, studies by Akhtar et al. (2019), Leach et al. (2017), and Qadri et al. (2020) suggest that combining multiple antigens—such as colonization factors CS5, CS3, and CFA/I—with cross-reactive antibodies effectively inhibits bacterial attachment in humans. This multi-antigen strategy provides a comprehensive defense against bacterial colonization and infection.

Beyond pharmaceutical interventions, bioactive compounds found in food, such as proanthocyanidins and polyphenols, have demonstrated the ability to bind bacterial flagella and pili, preventing aggregation and adhesion. This targeted approach offers advantages over conventional antibiotics, as it selectively disrupts bacterial adhesins without inducing bacterial death, thereby reducing the risk of resistance development (Gowd et al., 2019). While sub-inhibitory antibiotic doses can also reduce bacterial adherence, they contribute to the spread of antibiotic resistance genes (ARGs) due to selective pressure. In contrast, natural bioactive compounds present a promising early-stage therapy alternative, as they do not promote antibiotic-resistant bacteria (Gurumallu and Javaraiah, 2021). Continued research into adhesion inhibitors and their mechanisms of action holds great potential for novel therapeutic interventions against bacterial infections.

#### 4.1.3 T6SS and OMVs

The Type VI secretion system (T6SS) is a specialized mechanism employed by Gram-negative bacteria to inject toxic compounds into rival microbes, fostering microbial competition (Borgeaud et al., 2015). This system allows pathogens to bypass the natural defense mechanisms of the host’s commensal microbiota, granting them a competitive edge and enabling prolonged persistence within the host. Additionally, bacterial cell death triggered by T6SS releases DNA, serving as a substrate for horizontal gene transfer and facilitating the spread of antibiotic resistance genes (ARGs) (Hachani et al., 2016). By manipulating the microbiota in this manner, pathogenic bacteria can evade immune responses and outcompete beneficial microbes.

**Figure 6:**
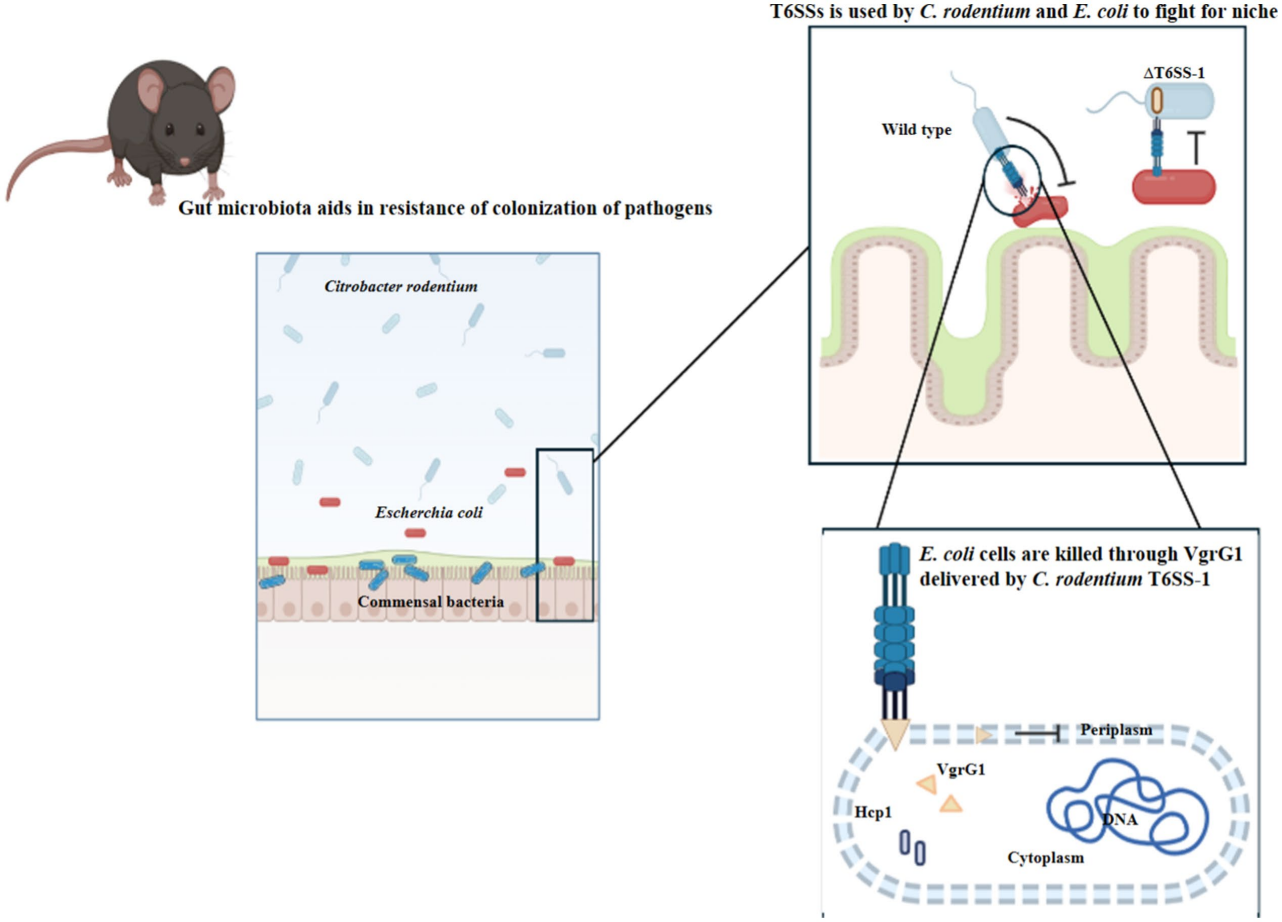
Pathogenic *Escherichia coli* (*E. coli*) and commensal *Citrobacter rodentium* (*C. rodentium*) employ Type VI secretion systems to facilitate their colonization and persistence within the gut environment. (Created with BioRender.com).

Outer membrane vesicles (OMVs) play a crucial role in bacterial competition, antibiotic resistance, and horizontal gene transfer. Recent findings by Li et al. (2022) highlight the importance of the lipopolysaccharide (LPS)-binding effector TeoL in T6SS-mediated recognition and utilization of OMVs. TeoL interacts with OMVs in the surrounding environment, enhancing bacterial adaptation. T6SS thus provides a substantial advantage for bacteria colonizing symbiotic microbiota niches (Figure 6), shaping microbial communities and influencing horizontal gene transfer. Exploring the interactions between T6SS, OMVs, and gene transfer mechanisms could provide deeper insights into the role of T6SS in the dissemination of ARGs.

Efficient suppression of the Type VI Secretion System (T6SS) presents a promising strategy to control the infiltration of symbiotic bacteria into specific niches and regulate the invasion of foreign pathogens. Research by Sima et al. (2018) and Balta et al. (2021) has demonstrated that Auranta 3,001, a formulation containing organic acids and plant extracts, effectively reduces the infection potential and cecal colonization of *Campylobacter coli* and *Campylobacter jejuni*. This occurs through the downregulation of genes associated with T6SS. Similarly, paramyxoviruses can diminish their virulence when exposed to a combination of citrus extract, lactic acid, and citric acid (E330), which inhibits the T6SS-associated *hcp1* and *hcp2* genes. These findings suggest that reducing T6SS activity or minimizing direct interactions with unrelated bacteria can help protect bacterial populations while preserving microbial diversity and abundance. By counteracting T6SS-dependent mortality, this approach enhances the resilience of commensal bacteria, enabling them to better withstand colonization in the human host.

### 4.2 Microbial colonization resistance

Colonization resistance (CR) is vital in host-microbial interactions. It actively hinders the establishment of foreign microbes in the body by many mechanisms, such as immune-mediated responses (Buffie and Pamer, 2013). The mechanisms involve the secretion of antimicrobial substances such as bacteriocins, bile acids, and short-chain fatty acids. They also include competing for necessary nutrients, maintaining the integrity of the intestinal barrier, and influencing the host’s immune response (Seekatz et al., 2022; Styczynski et al., 2023). Microbiota disruptions caused by variables, including antibiotic usage and non-antibiotic medications, such as antipsychotics and anti-diabetic drugs, might change the makeup of microbes, leading to a compromised ability to resist colonization (Seekatz et al., 2022).

The host’s microbiota typically demonstrates tolerance towards the majority of invading germs. Microbiomes with a higher diversity level show a more vital ability to withstand and recover from external bacterial assaults or infections. Hence, maintaining the composition and variety of indigenous microbiota improves its capacity to resist colonization by exogenous bacteria (Styczynski et al., 2023). ARGs can spread among bacterial species by horizontal gene transfer (HGT), particularly in diverse microbial communities like human and animal gut. Gaining a more profound comprehension of horizontal gene transfer (HGT) pathways in the gastrointestinal tract could expedite the advancement of medicines to diminish the dissemination of antibiotic-resistant genes (Ducarmon et al., 2019). Several studies (Buffie and Pamer, 2013; Khazaei et al., 2020; Le Guern et al., 2021) have confirmed a robust association between the distribution of ARGs and the evolutionary relationships of bacteria. In addition, the microbiome makeup is influenced by several factors that also impact the abundance of ARGs.

#### 4.2.1 Gut microbiome response to antibiotics

The human gut microbiome serves as a significant reservoir of antibiotic resistance genes (ARGs) and plays a crucial role in the emergence and spread of antibiotic-resistant bacteria (ARB) and ARGs (Zhang et al., 2024). Antibiotic use profoundly impacts the gut microbiome’s composition and resistance gene profile, often leading to the depletion of both beneficial and harmful bacteria. The extent of this impact depends on factors such as the specific antibiotic used, the mode of administration, and the patient’s preexisting gut microbiota (Ahuja et al., 2022). Broad-spectrum antibiotics, in particular, tend to reduce microbial diversity, facilitating the proliferation of antibiotic-specific ARGs. The influence of different antibiotics on the gut microbiome varies significantly. For instance, Willmann et al. (2019) demonstrated that cotrimoxazole administration led to a 148.1% increase in sulfonamide ARGs, whereas ciprofloxacin had little effect (Batra et al., 2017). Similarly, certain antibiotics, such as macrolides, glycopeptides, and *β*-lactams, can reduce populations of beneficial commensals like lactic acid bacteria and bifidobacteria, disrupting gut homeostasis (Li et al., 2023).

The distinction between bacteriostatic and bactericidal antibiotics further influences resistance development and dissemination. Bacteriostatic agents inhibit bacterial growth and replication without directly killing the bacteria, allowing the immune system to clear the infection. They primarily target processes like protein synthesis or metabolic pathways. In contrast, bactericidal agents directly kill bacteria by disrupting essential cellular structures, such as the cell wall, membrane, or DNA integrity (Hong P. Y. et al., 2020). The effectiveness of these agents depends on factors like bacterial species, antibiotic concentration, and environmental conditions. While bactericidal agents are often preferred for severe infections due to their rapid action, bacteriostatic agents are useful for controlling bacterial growth in less critical cases. Notably, bactericidal antibiotics tend to reduce mutation rates, thereby lowering the risk of resistance development.

The mode of antibiotic administration also influences gut microbiota disruption and ARG selection. Studies by Fan et al. (2021), Guo et al. (2022), Kelly et al. (2021), and Zhang et al. (2013) indicate that injectable tetracycline and ampicillin have a lesser impact on intestinal ARGs compared to oral administration. Injections help maintain microbiota stability and reduce the depletion of beneficial bacteria. Additionally, the composition of the gut microbiome before antibiotic treatment plays a crucial role in determining treatment outcomes. Interestingly, non-antibiotic drugs can also contribute to ARG dissemination in clinical and environmental settings through plasmid-mediated transfer (Ding et al., 2022; Wang M. et al., 2022).

Given these complexities, a comprehensive assessment of antibiotic administration’s effects on the microbiome and resistome in real-world scenarios is essential. Translating laboratory and clinical findings into practical strategies is key to combating antibiotic resistance while minimizing harm to the host. Selecting antibiotics and delivery methods that exert minimal impact on the gut microbiota is crucial. Advances in precision medicine, such as supramolecular antibiotics, gold nanoparticles, bacteriophage therapy, antibody-antibiotic conjugates, and nanoparticle-based drug delivery, offer promising solutions for targeted antimicrobial therapy. Maier et al. (2021) identified compounds like benzbromarone, dicumarol, and tolfenamic acid, which protect Bacteroides from erythromycin without affecting other microbes. Such counteractive agents, along with optimized drug administration techniques and the use of specific antibiotics, can significantly reduce microbiota disruption and limit ARG proliferation. By integrating these approaches, we can mitigate the adverse effects of antibiotics while maintaining a balanced and resilient gut microbiome.

#### 4.2.2 Nutritional strategies for gut health and antibiotic resistance

Maintaining a healthy gut microbiome is essential for defending against the invasion of harmful microorganisms. Diet plays a significant role in shaping the composition and resistance capabilities of the gut microbiota. Polyphenols, found in fruits, vegetables, cereals, tea, coffee, and wine, have garnered attention for their potential health benefits, including antioxidant, anti-inflammatory, and anticancer properties (de Llano et al., 2020; Gowd et al., 2019; Lal et al., 2021; Maisto et al., 2023). *In vitro* studies have shown that polyphenols can regulate the human gut microbiota by inhibiting harmful pathogens such as *Helicobacter pylori* and *Staphylococcus* sp., while promoting beneficial bacteria like *Lactobacillus* and *Bifidobacteria* (Gowd et al., 2019). Evidence from animal and clinical trials further supports that polyphenols influence gut microbial composition, diversity, and the Firmicutes to Bacteroidetes (F/B) ratio, largely due to their prebiotic-like effects (Man et al., 2020). Flavonoids such as anthocyanins, phenolic acids like epicatechins, p-coumaric acid, and o-coumaric acid, and other polyphenols, including quercetin, rutin, chlorogenic acid, and caffeic acid, have been shown to enhance beneficial gut bacteria like *Bifidobacterium* and *Lactobacillus* while reducing harmful bacterial colonization (Samarasinghe et al., 2019). Catechins, a subgroup of polyphenols, promote helpful bacteria such as *Clostridium coccoides*, *Eubacterium rectale*, and *Bifidobacterium* sp., while inhibiting harmful bacteria like *Clostridium histolyticum* (Kumar Singh et al., 2019).

However, antimicrobial treatments can disrupt the balance of gut bacteria, reducing beneficial species, particularly those that produce short-chain fatty acids (SCFAs). This imbalance can create a favorable environment for the growth of carbapenem-resistant *Enterobacteriaceae* (CRE) (Lagadinou et al., 2024; Korach-Rechtman et al., 2020; Sorbara et al., 2019). Additionally, diets high in sugar, fat, and protein have been linked to the promotion of skin bacteria, facilitating the spread of genes that confer antibiotic resistance. A study by Tan et al. (2022) found that dietary patterns could potentially enhance the expression of regulatory genes associated with the amplification and transfer of antibiotic resistance genes (ARG). Research has shown a strong correlation between high-fat diets and an increase in specific ARGs and mobile genetic elements (MGE), leading to dysbiosis and compromised immune function (Tan et al., 2022). Diets tailored to promote gut health, such as low-fat, high-fiber diets, can help maintain a healthy microbiome and prevent the development of antibiotic-resistant bacteria.

Further studies have shown that polyphenols, along with essential nutrients like vitamins A and D, and Omega-3 fatty acids, significantly impact gut microbiota and its barrier function (Cantorna et al., 2019; Duan et al., 2022; Gowd et al., 2019; Kawabata et al., 2013). Vitamin A, for instance, is suggested as an adjunctive treatment for infectious diseases and autism spectrum disorders (ASD), potentially by modulating the gut microbiota (Cantorna et al., 2019). Deficiency in vitamin A has been linked to reduced gut microbiota diversity and lower levels of butyrate-producing bacteria. On the other hand, supplementation with retinoic acid has been shown to inhibit murine Norovirus replication and lessen infection severity (Lee and Ko, 2016).

Higher calcium intake has been associated with a reduced prevalence of obesity, likely due to changes in gut flora linked to lean body composition (Surmeneva et al., 2019). Intervention trials have found that a daily calcium intake of 1,000 mg increases the presence of beneficial *Clostridium XVIII* in male fecal samples, indicating a higher abundance of butyrate-producing bacteria (Yang et al., 2020). In high-fat diet mouse studies, calcium supplementation at a dosage of 5.25 g/kg enhanced the diversity and abundance of *Ruminococcaceae* and *Akkermansia* bacteria in the fecal microbiome. Such interventions are essential to maintain the structural integrity of the intestinal lining and promote a healthy balance of beneficial bacteria, which is crucial for preventing the spread of harmful bacteria and antibiotic-resistant genes (Surmeneva et al., 2019). Non-nutritive sweeteners, as specific dietary additives, have antimicrobial properties and can mimic the effects of antibiotics on the gut microbiota (Yang et al., 2020). Therefore, it is critical to evaluate dietary choices and nutritional intake carefully to regulate the gut microbiome and its susceptibility to antibiotic resistance. More research is needed to determine how dietary interventions can mitigate health risks related to the load of ARGs and antibiotic resistance, as well as to identify which food components are most effective against different antibiotic-resistant bacteria (ARB).

### 4.3 Overcoming antibiotic resistance: reversing resistance and enhancing susceptibility

Bacteria have developed a variety of mechanisms, both inherent and acquired, to mitigate the adverse effects of antibiotics (Table 8). Intrinsic resistance predominantly relies on three principal strategies: enzymatic inactivation or alteration of antibiotics, mutation of antibiotic targets, and the presence of cellular membrane barriers and efflux pumps that restrict antibiotic penetration (Zhang H. et al., 2023; Zhang R. M. et al., 2023). Conversely, acquired resistance primarily results from horizontal gene transfer (HGT) (Chen et al., 2021). This process encompasses mechanisms such as transformation (incorporation of exogenous DNA into the bacterial genome), transduction (transfer of DNA mediated by bacteriophages), conjugation (direct exchange of genetic elements between bacteria), and DNA transfer facilitated by membrane vesicle transport (Bai et al., 2022). Developing these mechanisms enables bacteria to acquire antibiotic resistance, leading to the emergence of highly resistant strains. Carbapenemase-producing colistin-resistant *Klebsiella pneumoniae* is an appropriate example of this phenomenon because it exhibits resistance to almost all of the currently available antibiotics (Weterings et al., 2015). The World Health Organization (WHO) has expressed serious concern regarding a critical shortage of effective antibiotics and a concern about the lack of new research and development (R&D) efforts in the field (World Health Organization, 2024; Bull et al., 2020; Bertagnolio et al., 2024). To combat the escalating threat of antibiotic resistance and ensure the continued efficacy of existing antibiotics, strategies beyond the sole development of novel drugs are crucial.

**Table 8:** Comprehensive strategies and mechanisms for combating antibiotic resistance: approaches, examples, and applications (Gholipour et al., 2024; Gitaka et al., 2020; Kasimanickam et al., 2021; Pattnaik et al., 2023).

Strategy	Description	Examples
1. Antibiotic Adjuvants	Chemical compounds or molecules are administered alongside antibiotics to enhance their efficacy or counteract resistance mechanisms.	Example: Beta-lactamase inhibitors like clavulanic acid inhibit bacterial enzymes that degrade beta-lactam antibiotics, thus restoring antibiotic activity.
2. Combination Therapy	Simultaneous use of two or more antibiotics targeting different pathways or mechanisms within bacteria, synergistically enhancing antimicrobial activity and minimizing resistance development.	Example: Using a combination of beta-lactam antibiotics and beta-lactamase inhibitors to combat beta-lactamase-producing bacteria.
3. Bacteriophage Therapy	Using bacteriophages, viruses that infect and kill bacteria, to target specific antibiotic-resistant strains, offering a precise and alternative therapeutic approach.	Example: Phage therapy treats infections caused by A*RB* like MRSA (Methicillin-resistant *Staphylococcus aureus*).
4. CRISPR-Cas Systems	Application of CRISPR-Cas gene editing technology to selectively target and deactivate ARGs within bacterial genomes, effectively reversing resistance phenotypes.	Example: CRISPR-Cas9-mediated disruption of genes encoding antibiotic resistance mechanisms in bacteria, rendering them susceptible to antibiotics.
5. Antibiotic Cycling	Rotating or alternating the use of different antibiotics over time to prevent the emergence and spread of resistance, exploiting bacterial vulnerabilities during susceptible phases.	Example: Rotating the use of different classes of antibiotics in hospitals based on surveillance data to reduce selective pressure and resistance emergence.
6. Repurposing Existing Drugs	Identifying and utilizing non-antibiotic drugs with secondary antimicrobial properties or synergistic effects when combined with antibiotics, expanding treatment options, and overcoming resistance.	Example: Repurposing antipsychotic drug chlorpromazine, which has been found to potentiate the activity of certain antibiotics against resistant bacteria.
7. Probiotics and Prebiotics	Administration of beneficial bacteria (probiotics) or compounds promoting their growth (prebiotics) to restore or maintain a healthy gut microbiota can indirectly enhance antibiotic susceptibility.	Example: Probiotics containing Lactobacillus species can modulate gut microbiota and reduce colonization by A*RB*.
8. Targeting Biofilm Formation	Disruption of bacterial biofilms, complex communities of bacteria encased in extracellular matrix, using enzymes, surfactants, or antibiofilm agents, which can restore antibiotic susceptibility.	Example: Treatment with biofilm-disrupting enzymes like dispersin B or DNase to degrade biofilm matrix and enhance antibiotic penetration.
9. Immunomodulation	Modulation of the host immune response to enhance clearance of bacterial infections, reducing the reliance on antibiotics and potentially reversing resistance by eliminating bacterial populations.	Example: Administration of immunostimulatory compounds like interferons or monoclonal antibodies targeting bacterial surface antigens to enhance host immunity against infections.
10. Environmental Interventions	Implementing measures to reduce environmental reservoirs of antibiotic resistance, such as improved sanitation, waste management, and antimicrobial stewardship practices, can limit resistance spread.	Example: Restricting antibiotics in agriculture and promoting responsible antibiotic use in healthcare to minimize environmental contamination and resistance dissemination.

#### 4.3.1 Combination of antibiotics

Combination antibiotic therapy, commonly used in clinical practice, involves administering two or more antibiotics together to prevent bacterial resistance (Liu L. et al., 2021; Liu Y. et al., 2021). This approach offers advantages over monotherapy, including improved efficacy, broader bacterial coverage, and potentially fewer side effects (Eskenazi et al., 2022). Specifically, combinations of aminoglycoside antibiotics (AGAs), such as gentamicin, enhance treatment effectiveness, accelerate bacterial eradication, and combat antibiotic resistance through synergistic effects with *β*-lactam antibiotics. β-lactams facilitate the entry of AGAs into bacteria by causing non-lethal damage to the bacterial cell wall, amplifying their bactericidal activity (Wang N. et al., 2022). This strategy is particularly useful in treating severe hospital-acquired infections caused by multidrug-resistant organisms, including pneumonia and sepsis.

Azithromycin, a widely used macrolide with strong antibacterial properties and a long half-life, is effective when combined with AGAs like gentamicin, specifically in treating *Pseudomonas aeruginosa* infections. This combination allows for dose reductions of both antibiotics (Wang N. et al., 2022). Gentamicin enhances the bactericidal effect of azithromycin on both planktonic and biofilm cells, with notable success in treating genitourinary gonorrhoea. Additionally, antimicrobial peptides (AMPs), derived from plants and animals, play a crucial role as the body’s first line of defense against pathogens (Costa et al., 2021). With broad-spectrum resistance and rapid action, AMPs are emerging as promising antibacterial agents. Research shows that AMPs, like PMAP-36 or PRW4, when combined with gentamicin, produce synergistic effects against *Escherichia coli* and *Staphylococcus aureus* by disrupting the bacterial outer membrane, enhancing permeability, and facilitating gentamicin’s entry into the cytoplasmic membrane. AMPs also exhibit direct antibacterial activity through interactions with intracellular targets like DNA.

In treating sepsis and severe Pseudomonas infections caused by gram-negative bacteria, combination therapies involving beta-lactam antibiotics, aminoglycosides, and fluoroquinolones are common (Fish et al., 2002). Studies have also evaluated the combination of polymyxin B, doripenem, and rifampin against multidrug-resistant strains of *A. baumannii* and *K. pneumoniae* (Fish et al., 2002). However, clinical outcomes can vary, and the development of antibiotic resistance is influenced by factors such as drug interactions and the inadvertent selection of resistant bacterial subpopulations. Some studies suggest that combination therapy may contribute to the emergence of resistance, exacerbating the spread of resistant strains (Liu et al., 2020; Bassetti et al., 2022). The increasing prevalence of antibiotic resistance, coupled with the slow pace of new antibiotic discovery, highlights the need for innovative therapeutic strategies, focusing on next-generation antibiotics and alternative treatments. While combination therapy can enhance antimicrobial treatment efficacy, it may also lead to unintended consequences (Liu L. et al., 2021; Liu Y. et al., 2021). In some cases, antibiotics may interact antagonistically, reducing each other’s effectiveness. This is particularly concerning when drugs target the same bacterial pathways or have overlapping mechanisms of action (Thu et al., 2021). For instance, combining beta-lactams with tetracyclines can reduce bacterial cell wall synthesis, as beta-lactams inhibit cell wall formation, while tetracyclines prevent protein synthesis (Hamadamin et al., 2024). This may impair the bacteria’s ability to respond to the cell wall damage caused by beta-lactams, reducing treatment efficacy.

Combination therapy, commonly used to treat bacterial infections, offers benefits such as a broader activity spectrum and reduced resistance risk. However, it can pose challenges like reduced bioavailability and increased toxicity (Stielow et al., 2023). When multiple drugs are combined, one may interfere with the absorption or distribution of another, decreasing its effectiveness. For instance, rifampin can reduce the bioavailability of protease inhibitors by inducing liver enzymes that accelerate their metabolism, lowering plasma concentrations (Zhuang et al., 2022). Additionally, combining drugs with overlapping toxicities, such as aminoglycosides and vancomycin, can increase nephrotoxicity and ototoxicity risks. Hence, carefully evaluating the risks and benefits of combination therapy is crucial to avoid reducing therapeutic efficacy or causing harmful side effects. While combination therapies are widely used to treat bacterial infections, they can lead to adverse effects, particularily in patients with impaired organ function or those on multiple medications (Thu et al., 2021). Antibiotics with different mechanisms of action may increase resistance, as seen in the use of ampicillin and ceftriaxone for *Enterococcus faecalis* infections, which has led to vancomycin-resistant enterococci colonization (Cusumano et al., 2022). Research by Okoliegbe et al. (2021) showed that combination therapy can enhance bacterial fitness and resistance, particularly through mexR gene inactivation in *P. aeruginosa*, which reduces antibiotic susceptibility. Mathematical models by Berríos-Caro et al. (2021) also demonstrated how resource scarcity and bacterial competition could drive resistance expression, resulting in double resistance under certain conditions.

The synergy in antimicrobial resistance (AMR) can have contradictory effects, accelerating pathogen elimination while promoting single-drug resistance. Carolus et al. (2024) found that collateral sensitivity could alter AMR development, potentially reducing resistance evolution. Anusha et al. (2023) highlighted that genetic evolution and cross-resistance are key when considering combination therapies. The development of resistance depends on factors like drug interactions and both host and pathogen characteristics. Angst et al. (2021) suggested that combination therapies are more effective than monotherapies if double resistance does not emerge, as resistance mutations usually occur independently, and fitness costs of resistance-associated genes are influenced by microbial competition. Future research should focus on understanding the mechanisms of resistance in combination therapies and exploring new combinations that minimize resistance development (Wang et al., 2022). Identifying synergistic effects and aligning treatments with the pathogen’s behavior can optimize therapeutic outcomes. Future studies should assess the impact of these strategies on clinical endpoints, such as infection resolution, patient morbidity, and long-term resistance patterns (Liu L. et al., 2021; Liu Y. et al., 2021; Hamadamin et al., 2024).

#### 4.3.2 Antibiotic-adjuvants

The notion of “adjuvant therapy” holds promise in antimicrobial therapies, yet its exploration remains constrained (Douafer et al., 2019). Thus, it is imperative to reassess the reliance solely on antibiotics for treating bacterial infections and instead explore non-antibiotic substances that could mitigate the emergence of antibiotic resistance (Douafer et al., 2019). Antibiotic adjuvants are compounds designed to bolster the effectiveness of antibiotics by either reducing or directly inhibiting mechanisms that confer resistance to them. The concept of antibiotic adjuvants draws from the successful use of synergistic combinations of two or more antibiotics in clinical settings (Hu Y. et al., 2023). These approaches, informed by empirical evidence, aim to harness the combined effects of multiple agents, broaden the spectrum of efficacy, and overcome resistance mechanisms. Unlike conventional antibiotic combinations, antibiotic adjuvants exhibit minimal or no antibacterial activity.

**Figure 7:**
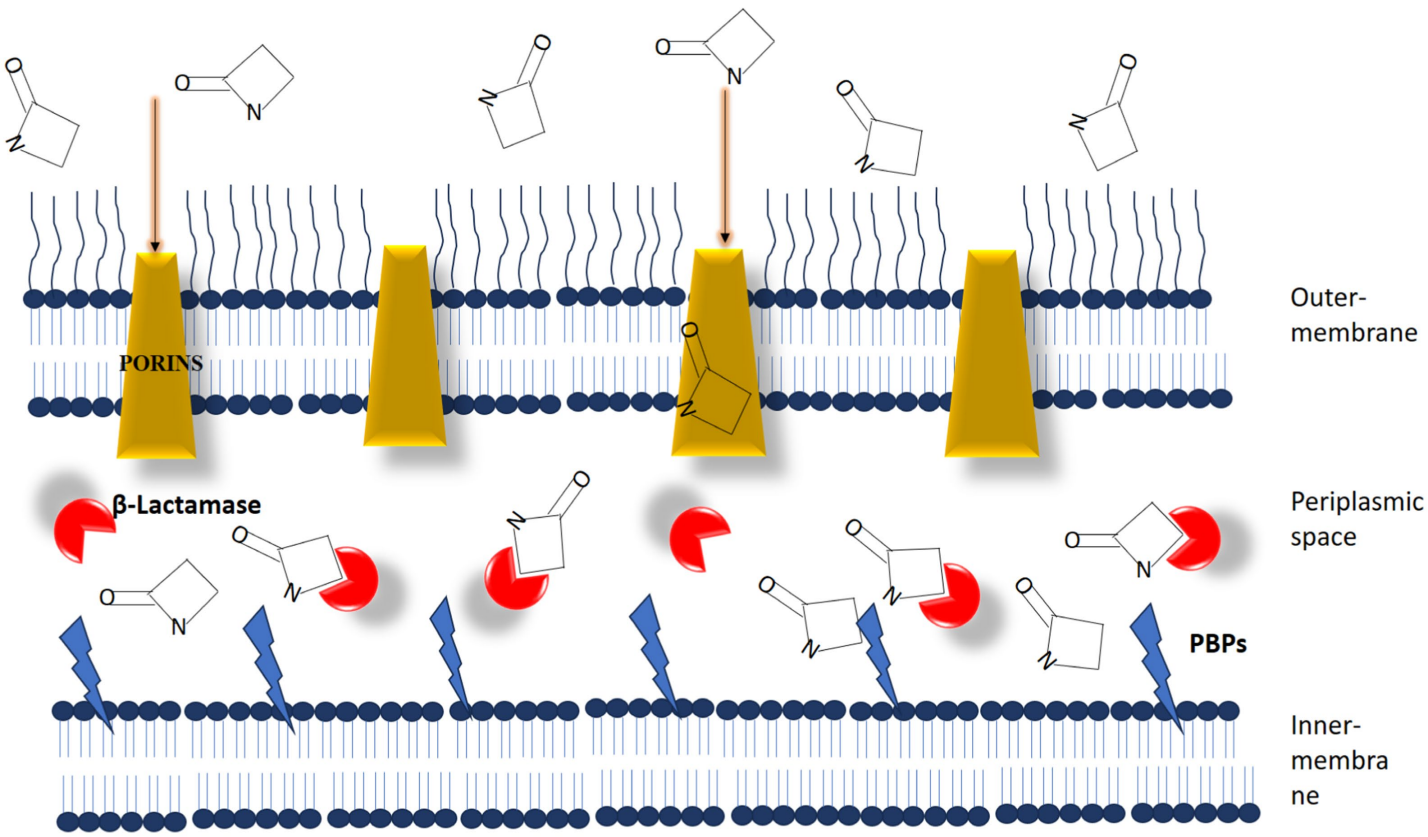
The dynamic interaction between β-lactam antibiotics and β-lactam interactive proteins in gram-negative bacteria plays a crucial role. β-lactamases function as interceptors, impeding the efficient binding of antibiotic molecules to penicillin-binding proteins (PBPs). [Source: Bush K. and Bradford P.A./Clinical Microbiology Reviews, 2020].

Antibiotic adjuvants can be classified into three primary types based on their target profiles: direct, indirect, and host-modulating resistance breakers (Douafer et al., 2019; Song et al., 2020). Direct antibiotic adjuvants target various active and passive resistance mechanisms in bacteria. These mechanisms fall into three main categories: outer membrane permeabilizers, efflux pump inhibitors, and *β*-lactamase inhibitors (Figure 7). β-lactamase inhibitors, such as diazabicyclooctanones (DBOs) and boronate-based compounds, have garnered attention for enhancing the efficacy of β-lactam antibiotics (Farhat and Khan, 2022). However, the emergence of reduced Susceptibility to DBOs, mainly due to KPC mutations, underscores the need for ongoing exploration of novel combinations. Efflux pump inhibitors (EPIs) that target specific pumps, such as NorA, AcrAB-TolC, and MexAB-OprM, have shown promise in combating multidrug resistance, as evidenced by studies conducted by Brawley et al. (2022), Tsutsumi et al. (2019), and Sulavik et al. (2001). Notwithstanding extensive investigation, none of these experimental pharmaceutical interventions (EPIs) have progressed to clinical utilization.

Polymyxins, surfactants, antimicrobial peptides, and other outer membrane permeabilizers make it easier for antibiotics to enter bacteria by making their membranes more permeable. Studies by researchers (Song et al., 2020; Song et al., 2022; Song et al., 2023) have validated the efficacy of SLAP-S25 and nordihydroguaiaretic acid (NDGA) as broad-spectrum antibiotic adjuvants. Combining adjuvants with antibiotics represents a proactive strategy against multidrug-resistant (MDR) pathogens. These combinations augment the effectiveness of existing antibiotics and help forestall the development of novel antibiotic resistance mechanisms.

#### 4.3.3 Bacteriophage therapy

**Figure 8:**
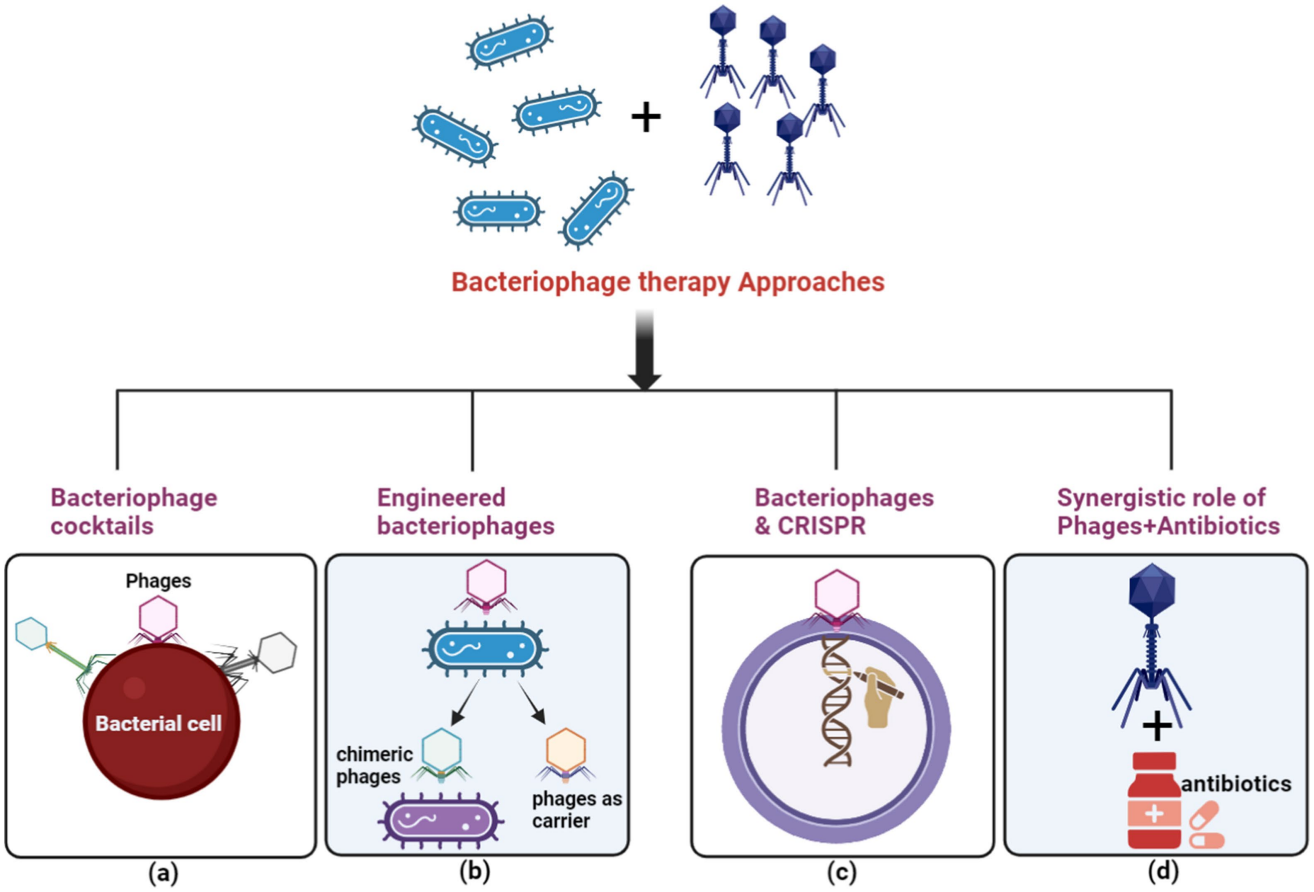
The principal methods for executing phage therapy include. **(A)** Utilizing a variety of bacteriophages in a phage cocktail to selectively target ARB. **(B)** Employing bacteriophage engineering to augment the efficacy of the response against ARB. **(C)** Employing bacteriophages to deliver the clustered regularly interspaced short palindromic repeats-associated (CRISPR-Cas) system for host cell elimination. **(D)** Depicting the synergistic effect between bacteriophages and antibiotics with a green circle symbol. (Created with BioRender.com).

Highly specific for their bacterial targets, bacteriophages (phages) have emerged as a promising alternative therapeutic strategy, particularly for combating multidrug-resistant (MDR) bacteria (Moghadam et al., 2020) (Figure 8). While introducing antibiotics initially overshadowed Felix d’Herelle’s pioneering discovery of phages in 1910 (Vandamme and Mortelmans, 2019), the alarming rise of antibiotic resistance has reignited interest in phage therapy. Unlike broad-spectrum antibiotics, phages exhibit remarkable selectivity, lysing only targeted bacterial strains, including MDR pathogens, while preserving the commensal microbiota. This unique characteristic has spurred exploring various phage-based therapeutic approaches, including phage cocktails, combination therapies with antibiotics, and genetically modified phages (Moghadam et al., 2020; Yacoby et al., 2007). Phage cocktails, offering a broader antimicrobial spectrum, have demonstrated superior efficacy in eradicating bacterial populations compared to single-phage therapies, potentially reducing the emergence of resistance.

Clinical successes have been documented in the treatment of *Mycobacterium abscessus* infections in cystic fibrosis patients and *Pseudomonas aeruginosa* infections in burn victims (Dedrick et al., 2023). Despite these achievements, significant challenges remain in the use of phage cocktails for antimicrobial therapy (Kering et al., 2019). Key areas of ongoing research focus on identifying optimal multi-component mixtures that target specific pathogens and expanding the host range of phages. Additionally, the co-evolution of bacteria and phages requires continual efforts to address the emergence of phage resistance within bacterial populations.

Phage-antibiotic synergy (PAS) presents a promising approach to enhance antibacterial efficacy while mitigating resistance development (Comeau et al., 2007). PAS exploits the phenomenon whereby sub-inhibitory antibiotic concentrations can promote phage replication, leading to a synergistic reduction in bacterial populations. Combining phage therapy with antibiotics has shown promise in treating infections caused by multi-drug resistant (MDR) bacteria, including MRSA and pan-drug-resistant *Klebsiella pneumoniae* (Eskenazi et al., 2022; Zalewska-Piątek and Piątek, 2020). However, it is crucial to carefully assess each phage-antibiotic combination, as not all are synergistic; some antibiotics may inadvertently hinder phage replication and reduce their effectiveness.

Bioengineered phages offer exciting possibilities for enhancing antibiotic efficacy and specifically targeting MDR bacterial infections (Cui et al., 2023). These phages can be engineered in various ways, such as increasing host diversity, altering host specificity, delivering foreign genes, and modifying capsids. For instance, modifying phages to deliver antibiotics directly into bacterial cells has led to substantial therapeutic improvements (Kumar et al., 2023). Additionally, the use of CRISPR-Cas systems to target virulence factors and antibiotic resistance genes (ARGs) in bacteria shows promise for restoring antibiotic susceptibility in resistant strains (Wu et al., 2021). By introducing the long tail fibre gene from bacteriophage IP008 into T2 bacteriophage, the host range of *E. coli* can be broadened (Dunne et al., 2021). Pires et al. (2016) developed a hybrid bacteriophage that combines IP008’s broad host range with T2’s potent cell destruction capability. Similarly, Tabib-Salazar et al. (2017) modified *E. coli* bacteriophage T7 to produce enzyme protein B, which disrupts biofilms and enhances host cell invasion, reducing cellular biofilms by over 100-fold.

Moreover, bioengineered bacteriophages can improve antibiotic selectivity and address the challenge of multidrug-resistant infections. A pediatric cystic fibrosis patient with a life-threatening infection from antibiotic-resistant *Mycobacterium abscessus* was successfully treated using a combination of three genetically modified bacteriophages after conventional treatments had failed (Adesanya et al., 2020). This novel treatment led to a favorable prognosis without significant adverse effects. Bacteriophages are increasingly being used as theranostic platforms, with advancements in synthetic biology and drug delivery strategies. For instance, Yacoby et al. (2007) developed a delivery method that attaches chloramphenicol molecules to lysed bacteriophages, resulting in a 2000-fold increase in treatment efficacy while reducing side effects. Furthermore, phages can be employed to transport photosensitizers for more efficient photodynamic inactivation of harmful bacteria, minimizing damage to the normal microbial community (Sheng et al., 2022). *In vivo* studies have shown the effectiveness of this method in treating infections caused by antimicrobial-resistant bacteria and *Candida albicans*.

Despite these advancements, some bacteria have developed resistance to bacteriophages through mechanisms such as CRISPR-Cas immunity, restriction-modification systems, and receptor mutations that prevent phage attachment (Hasan and Ahn, 2022). For example, *Escherichia coli* can acquire CRISPR-Cas spacers to recognize and degrade phage DNA, conferring resistance to certain bacteriophages (Mitić et al., 2023). To overcome these challenges, researchers are developing genetically engineered bacteriophages that bypass bacterial defense mechanisms, enhance phage lysis of resistant bacteria, and deliver antimicrobial genes. Ongoing research, including the engineering of Mycobacteriophage D29 to target antibiotic-resistant *Mycobacterium tuberculosis*, suggests that these phages could provide an alternative to traditional antibiotics in combating antimicrobial resistance (Pal et al., 2024; Biswas et al., 2024).

Bacteriophage-based technology also has significant potential in the field of cancer treatment. By displaying antibodies, peptides, or proteins on the surfaces of bacteriophages, researchers can utilize them to study the molecular characteristics of tumor cells and the tumor microenvironment. Phages serve as effective delivery vehicles for imaging agents and therapeutic treatments, making them valuable tools in tumor immunology. Additionally, phages have been engineered to deliver suicide genes into cancer cells, significantly improving the efficacy of gene therapy for anti-tumor treatments. Bioengineered bacteriophages hold great promise across a variety of biomedical applications. However, further research is needed to fully exploit their therapeutic potential and to overcome the challenges posed by bacterial resistance mechanisms.

#### 4.3.4 Monoclonal antibodies and vaccines

Due to their targeted action, monoclonal antibodies (mAbs) exhibit promise in treating severe bacterial infections (Imran et al., 2021). Clinical investigations are underway for humanized mAb CMTX-001, which targets the DNABII protein crucial for biofilm formation, thereby enhancing antibiotic efficacy and bacterial eradication by disrupting biofilms (Ding et al., 2023).

**Figure 9:**
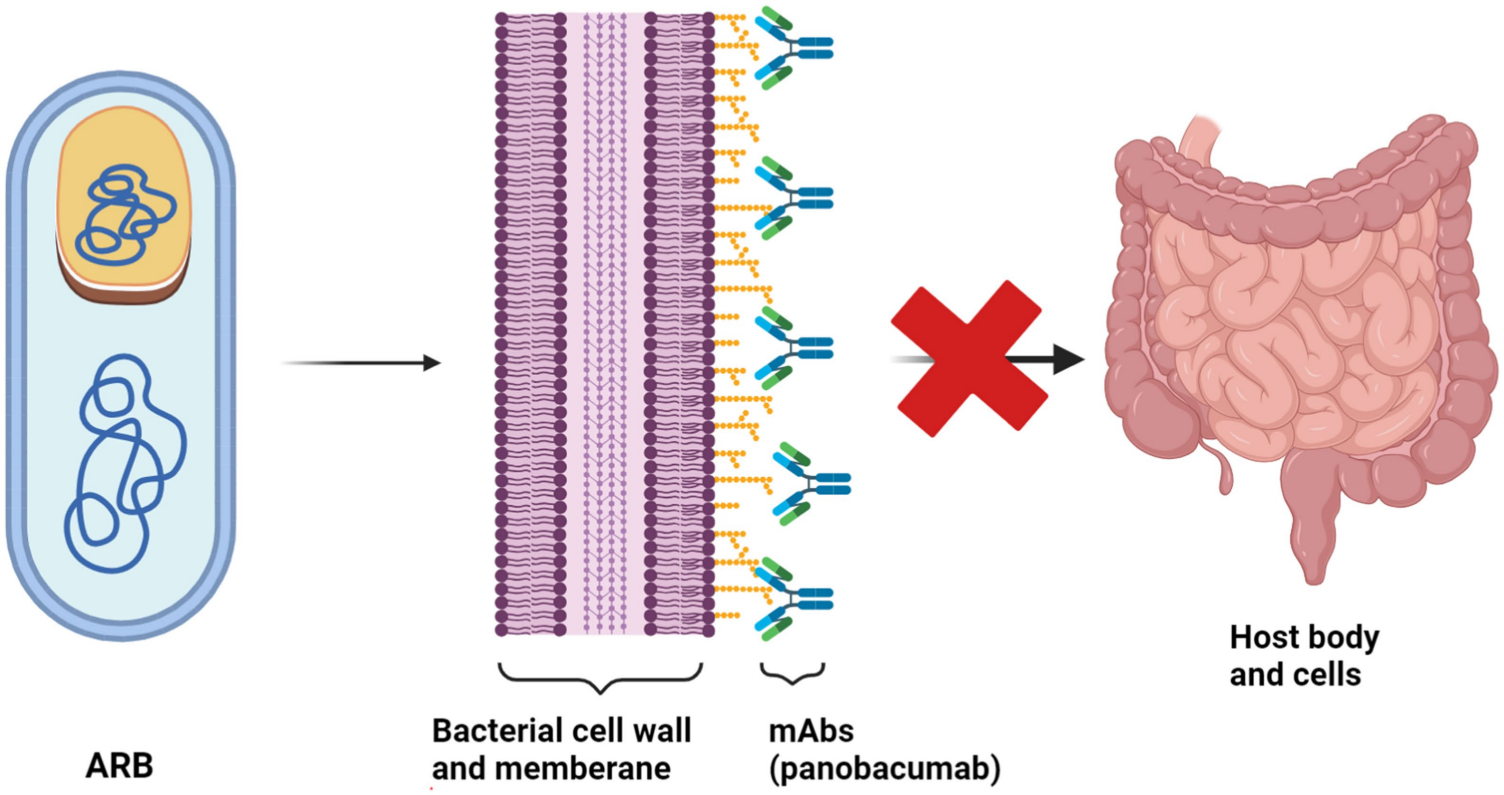
The mechanism of action of Panobacumab monoclonal antibody against *Pseudomonas aeruginosa* Panobacumab operates through a unique mechanism to combat infections caused by *P. aeruginosa*, a notorious pathogen known for its resistance to multiple antibiotics. This monoclonal antibody targets a specific antigen on the surface of *P. aeruginosa* bacteria, namely, the PcrV protein, a critical component of the type III secretion system (T3SS). (Created with BioRender.com).

Moreover, mAbs can target bacterial virulence factors. For instance, panobacumab neutralizes the outer membrane and lipopolysaccharide (LPS) of *Pseudomonas aeruginosa* (Figure 9), while the KB001 fusion antibody shows potential in targeting bacterial virulence factors (Nagy et al., 2017). In spite of their therapeutic potential, the development and clinical utilization of mAbs are challenging due to high production costs and the need for intravenous administration.

In addition to combating bacterial infections, vaccines play a pivotal role in reducing antibiotic usage and resistance. The success of *Streptococcus pneumoniae* vaccination has reduced antibiotic-resistant pneumococcal strains and antibiotic consumption by bolstering herd immunity (Saeed et al., 2023). Similarly, the introduction of conjugated *Haemophilus influenzae* type b (Hib) vaccinations has substantially decreased Hib-related illnesses and beta-lactamase-producing strains, thus mitigating antibiotic resistance (Storz et al., 2016). These achievements underscore the importance of ongoing vaccine research in combating bacterial diseases. While both vaccines and mAbs offer the potential to fight bacterial infections, mAbs face challenges related to cost and administration (Imran et al., 2021). Conversely, preventive vaccines can potentially significantly decrease antibiotic use and resistance. A comprehensive and practical approach to bacterial pathogens necessitates continued research and development in vaccine and mAb domains.

Regardless of promising findings in treating multidrug-resistant (MDR) bacterial infections, mAb-based immunotherapy encounters significant development hurdles (Seixas et al., 2022). Challenges include the potential for humanized mAbs to elicit a human anti-chimeric antibody (HACA) response, rendering therapeutic efficacy uncertain (Almagro et al., 2018). Moreover, mAb targets are often specific to bacterial antigens, emphasizing the importance of rapid pathogen detection. Additionally, mAbs may be less effective if the target antigen is expressed only in certain circulating strains, specific organ infections, or disease phases. Exopolysaccharide architectures, particularly serotype-specific variations, pose challenges for mAb efficacy against diverse bacterial strains. For instance, *Streptococcus pneumoniae* serotypes exhibit differences in exopolysaccharide capsule content and structure, complicating mAb efficacy across all serotypes (Wang-Lin and Balthasar, 2018).

Furthermore, PNAG (*β*-1-6-linked poly-N-acetyl-d-glucosamine), a conserved exopolysaccharide in numerous pathogens, influences microbial survival and biofilm formation (Champion et al., 2019). Although mAb F598 effectively minimized microbial issues across various models and microorganisms, further clinical studies were not pursued. Addressing commercial hurdles and aligning development efforts with disease market size is imperative for advancing broad-spectrum mAb development. Future research endeavours are warranted to address these challenges comprehensively.

#### 4.3.5 Nanoparticles

**Figure 10:**
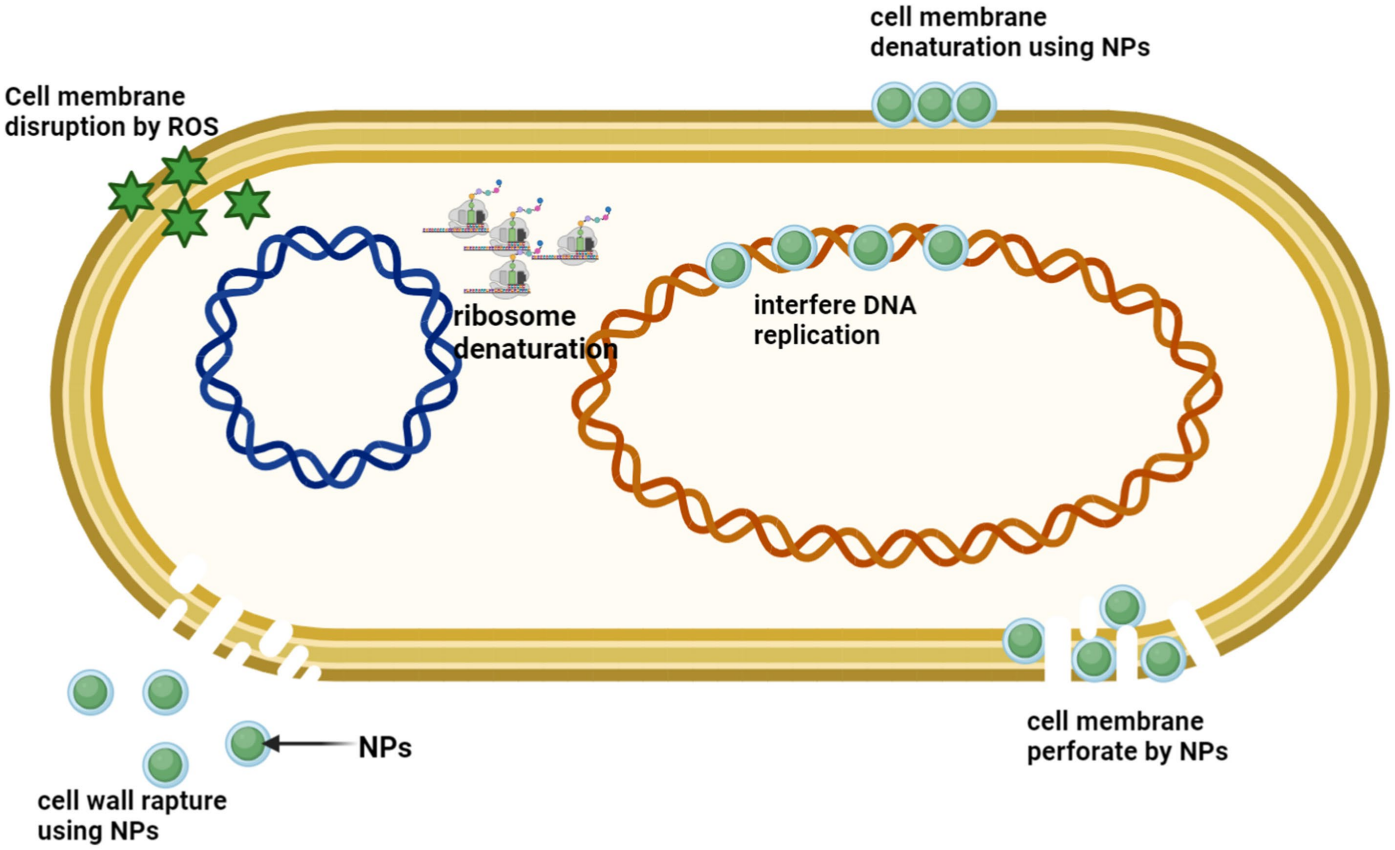
Silver nanoparticles (AgNPs) exhibit a range of antimicrobial properties through multiple mechanisms, including disruption of the cell wall and cytoplasmic membrane, denaturation of ribosomes, generation of reactive oxygen species (ROS) leading to membrane disruption, interference with DNA replication, and membrane denaturation and perforation. (Created with BioRender.com).

The combination of antibiotics with nanomaterials, particularly silver nanoparticles (AgNPs), has a combinatorial antibacterial effect (Figure 10) (Scandorieiro et al., 2022). Antibiotics commonly promote bacterial cell death by stimulating the generation of reactive oxygen species (ROS) (Hong Y. et al., 2020). Studies show that gentamicin significantly enhances the production of reactive oxygen species (ROS) by silver nanoparticles (AgNPs) (Mazur et al., 2020). Luminol chem-iluminescence (CL) indicates that reactive oxygen species (ROS) are generated when Tween-stabilized AgNPs have an antibacterial impact (Wang N. et al., 2022). The concurrent use of gentamicin and Tween-stabilized AgNPs exhibits a synergistic effect in combating gentamicin-resistant *Staphylococcus epidermidis*, as observed in the study by Mazur et al. (2020). The combination of AgNPs plus antibiotics can efficiently eradicate microbes through numerous pathways, enhancing their antibacterial effectiveness (Mohammed and Abdel Aziz, 2019; Scandorieiro et al., 2022). Silver nanoparticles (AgNPs) have been reported to infiltrate the bacteria’s cell wall, disrupt the cellular membrane, and trigger bacterial death. The study by Katva et al. (2017) revealed a noteworthy synergistic antibacterial impact when combining various antibiotics and AgNPs, specifically gentamicin and chloramphenicol, with AgNPs in the context of *E. faecalis* (Ef) infection (Katva et al., 2017).

Graphene is a well-known substance that is compatible with living organisms and has several uses in the fields of antibacterial activity, biosensing, cancer therapy, and biocarriers (Hu X. et al., 2023; Huang et al., 2022; Saha, Visconti, Desipio, & Saha et al., 2020; Zhang H. et al., 2023). The study (Wang N. et al., 2022) The study investigated the antibacterial properties of TOB-GO-Ag, a water-soluble hybrid composed of silver nanoparticles (AgNPs), graphene oxide (GO), and tobramycin,. The investigation focused on its effectiveness against multidrug-resistant gram-negative *E. coli* bacteria. The results revealed that TOB-GO-Ag demonstrated the highest antibacterial efficacy compared to GO, AgNPs, and tobramycin, which were used separately. Scientists (Wang N. et al., 2022) revealed how the TOB-GO-Ag composite fights bacteria. The combination disrupts the bacterial cell wall, allowing silver ions (Ag+) and graphene oxide (GO) to enter the cells. This one-two punch triggers oxidative stress within the bacteria, ultimately leading to their death. Additionally, the tobramycin component further hinders bacterial growth by blocking protein-synthesis (Ullah et al., 2018). Some compounds containing bismuth (Bi) have synergistic antibacterial properties when combined with antibiotics (Keogan and Griffith, 2014). Studies (Ma et al., 2017) have demonstrated that Bi2S3 nanoparticles do not possess antibacterial properties when tested against *Staphylococcus aureus* and methicillin-resistant *S. aureus* (MRSA), with minimum inhibitory concentration (MIC) values exceeding 1,024 μg/mL. Nevertheless, combining gentamicin with Bi2S3 nanoparticles demonstrates a synergistic antibacterial impact on MRSA. The reaction between Bi2S3 and gentamicin is the key strategy for this interaction. It leads to bacterial cell membrane rupturing, increased gentamicin accumulation, and the production of reactive oxygen species (ROS) (Ma et al., 2017).

CCNPs, or microscopic calcium carbonate particles, possess a consistent structure consisting of crystal grains measuring around 62.5 nanometers in diameter. Research has demonstrated that these CCNPs can function as carriers for the antibiotic gentamicin, effectively prolonging its release duration by up to 24 h. In addition, CCNPs significantly enhance the bactericidal efficacy of gentamicin (Pan et al., 2018). According to Pan et al. (2018), examining zeta potential and microscopic observations have shown that when CCNPs are absorbed into bacterial surfaces, they cause more harm to the bacterial cell wall and make the membrane more permeable. This ultimately results in more bacterial death.

#### 4.3.6 Fecal microbiota transplantation (FMT)

Fecal microbiota transplantation (FMT) has emerged as a promising intervention for addressing a range of gastrointestinal disorders, with potential applications in reducing the impact of antimicrobial resistance (AMR) (Lou et al., 2023; Singh et al., 2022). AMR, where bacteria evolve to resist the effects of antibiotics, poses a significant threat to global health by rendering many existing antibiotics ineffective. The rise of resistant infections is exacerbated by the overuse and misuse of antibiotics in human and veterinary medicine. FMT, a procedure that involves transplanting fecal bacteria from a healthy donor into the gastrointestinal tract of a recipient, has gained attention as a potential tool in the fight against AMR (Wang et al., 2021). The rationale behind FMT’s possible role in reducing AMR lies in its ability to restore a healthy, diverse microbiome, which can be crucial in preventing infections and reducing the need for antibiotics. Antibiotic resistance worsens outcomes in cirrhosis, and fecal microbiota transplant (FMT) may reduce antibiotic resistance gene (ARG) burden. The study bt Bajaj et al. (2021) analyzed ARG abundance in cirrhotic patients undergoing capsule or enema FMT across two trials. Capsule FMT reduced beta-lactamase and rifamycin ARGs, while enema FMT initially increased ARGs post-antibiotics but decreased them by day 15. Overall, FMT lowered ARG abundance compared to baseline and non-FMT groups, showing its potential in decompensated cirrhosis. In another study Rashidi et al. (2024) conducted a randomized placebo-controlled trial analyzed 226 stool samples from 100 patients undergoing intensive cancer therapy to assess the short- and long-term effects of fecal microbiota transplantation (FMT) on antibiotic resistance genes (ARGs). Initially, low-level transfer of ARGs from donor microbiota occurred, followed by long-term resistance to new ARGs as stable microbial communities formed. This suggests FMT may aid in reducing multidrug-resistant organism colonization in high-risk patients. Further research is needed to determine its clinical implications, particularly in preventing infections during intensive therapy. Woodworth et al. (2023) reported that Faecal microbiota transplantation (FMT) is a promising strategy for decolonising multidrug-resistant organisms (MDROs) like vancomycin-resistant enterococci (VRE) and carbapenemase-producing Enterobacteriaceae (CPE). This study assessed the genetic response of MDROs to FMT in 29 patients, showing a significant decrease in resistance gene expression, particularly VanA and blaNDM. Both culture-dependent and independent methods confirmed gene downregulation over time. FMT was well tolerated, with no adverse events, highlighting its potential for reducing MDRO carriage in infected patients.

FMT works by reintroducing a balanced microbiome into a recipient’s gut, often after it has been disrupted by factors such as antibiotic use, disease, or other environmental factors. The gut microbiome is a complex ecosystem of bacteria, viruses, fungi, and other microorganisms that play a vital role in digestion, immune function, and protection against pathogenic microbes (Bajaj et al., 2021). When this microbiome is disrupted, often by antibiotics, the balance of microorganisms in the gut is disturbed, allowing pathogenic bacteria, including antibiotic-resistant strains, to proliferate. By restoring this balance, FMT can help re-establish microbial communities that outcompete harmful pathogens, including antibiotic-resistant ones, and may reduce the need for further antibiotic treatment (Minkoff et al., 2023). One of the most well-established uses of FMT is in treating *Clostridium difficile* infection (CDI), a common and serious bacterial infection often triggered by antibiotic use (Gupta et al., 2022; Nooij et al., 2021). Antibiotic treatments for CDI can disrupt the gut microbiome, leading to recurrent infections. FMT is highly effective in treating recurrent CDI, with up to 90% success rates. This success is largely attributed to the ability of FMT to restore a healthy microbiome that can prevent the overgrowth of *C. difficile* (Gupta et al., 2022). This microbiome restoration helps control CDI and reduces reliance on antibiotics, which is crucial in the fight against AMR.

FMT also promises to reduce the colonization and transmission of multidrug-resistant organisms (MDROs). Studies (Nooij et al., 2021; Chiu et al., 2021) have shown that restoring a healthy gut microbiota through FMT can reduce the colonization of resistant bacteria, such as extended-spectrum beta-lactamase (ESBL)-producing *E. coli* and vancomycin-resistant Enterococcus (VRE), in patients who are carriers of these pathogens. The process works by rebalancing the microbiota and restoring microbial diversity, which can help limit the dominance of resistant bacteria, potentially reducing their spread within healthcare settings.

Furthermore, FMT may contribute to reducing AMR by promoting the natural resilience of the microbiome to pathogenic and resistant bacteria. A healthy microbiome is a barrier to pathogenic organisms by outcompeting them for resources and producing substances that inhibit their growth. This resilience could help prevent infections from developing or becoming severe, thus reducing the need for antibiotics and limiting the opportunity for the emergence of resistance (Bajaj et al., 2021). While FMT shows significant promise in addressing AMR, its widespread use still has challenges and limitations. Standardizing FMT protocols, ensuring the safety and quality of donor samples, and understanding the long-term effects of microbiome restoration are all areas that require further research.

Additionally, FMT may not be appropriate for all patients, particularly those with compromised immune systems or severe underlying health conditions (Lou et al., 2023). Fecal microbiota transplantation represents a novel and promising approach to reducing the impact of AMR. By restoring a healthy microbiome, FMT can lessen the prevalence of antibiotic-resistant infections, lower the need for antibiotics, and potentially prevent the spread of resistant pathogens (Wang et al., 2021). As more research is conducted, FMT may become an integral part of strategies to combat AMR, providing a valuable alternative to traditional antibiotic treatments and preserving antibiotic efficacy for future generations.

## 5 Conclusion and outlook

In conclusion, the COVID-19 pandemic has highlighted the urgent need to tackle bacterial resistance as a global health threat. Despite a decrease in global antibiotic consumption, the overall volume remains high, underscoring the need for stricter regulations and proactive prevention strategies. Although National Action Plans (NAPs) have been implemented worldwide, significant disparities persist, specifically in developing countries where limited resources and healthcare infrastructure hinder effective action. Key reservoirs for antibiotic-resistant bacteria (ARB) and resistance genes (ARGs) include farms, hospitals, wastewater treatment plants, and agricultural settings, necessitating targeted mitigation strategies.

While promising approaches such as dietary modifications, probiotic supplementation, and combination therapies with adjuvants or phages offer potential, challenges remain in their large-scale implementation, regulatory approval, and long-term effectiveness. Dietary modifications may be ineffective if they do not specifically target pathogenic resistance mechanisms or modify the microbiome in beneficial ways. Probiotics may struggle to outcompete resistant bacteria, specifically in severe cases of antimicrobial resistance (ABR). Combination therapies could also face resistance if pathogens evolve against phages or adjuvants, or if biofilm formation or immune system interference hinders phage efficacy.

Emerging treatments like customized antibiotics, monoclonal antibodies, vaccines, and nanoparticles offer alternatives but face challenges in cost, technological complexity, and accessibility. Despite advances in next-generation sequencing and bioinformatics, there are still gaps in understanding the role of gut microbiota in resistance dynamics, indicating a need for further research. Effectively addressing antibiotic resistance requires comprehensive policies and regulatory frameworks that balance public health protection with sustainable antibiotic use. However, global coordination remains challenging due to differences in governance, economics, and healthcare priorities. Strengthening surveillance, raising public awareness, and fostering interdisciplinary collaboration will be key to overcoming these barriers. Through evidence-based strategies and global cooperation, we can work toward managing antibiotic resistance and ensuring the continued efficacy of antibiotics for future generations.
